# Complex Nanoparticle Diffusional Motion in Liquid-Cell Transmission Electron Microscopy

**DOI:** 10.1021/acs.jpcc.0c03203

**Published:** 2020-06-10

**Authors:** Evangelos Bakalis, Lucas R. Parent, Maria Vratsanos, Chiwoo Park, Nathan C. Gianneschi, Francesco Zerbetto

**Affiliations:** † Dipartimento di Chimica “G. Ciamician”, Universita di Bologna, V. F. Selmi 2, 40126 Bologna, Italy; ‡ Innovation Partnership Building, The University of Connecticut, Storrs, Connecticut 06269, United States; § Department of Materials Science & Engineering, Northwestern University, Evanston, Illinois 60208, United States; ∥ Department of Industrial and Manufacturing Engineering, Florida State University, Tallahassee, Florida 32306, United States; ⊥ Department of Chemistry, Department of Materials Science & Engineering, and Department of Biomedical Engineering, Northwestern University, Evanston, Illinois 60208, United States

## Abstract

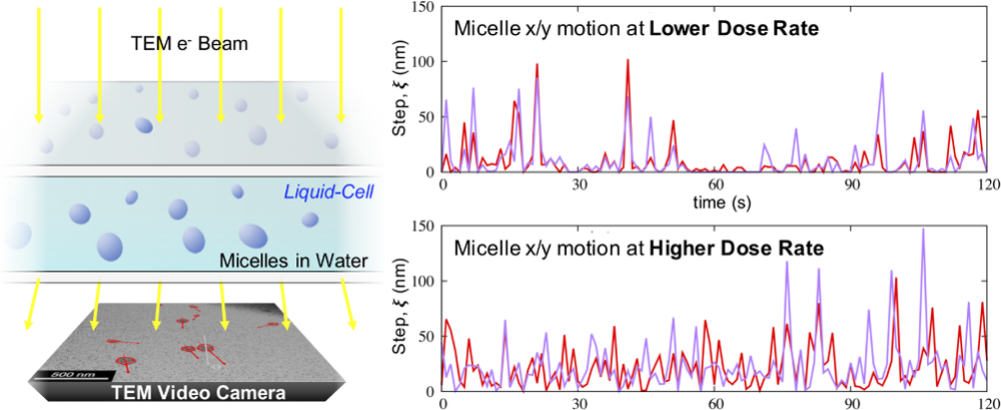

Liquid-cell transmission electron microscopy (LCTEM) is a powerful in situ videography technique that has the potential to allow us to observe solution-phase dynamic processes at the nanoscale, including imaging the diffusion and interaction of nanoparticles. Artefactual effects imposed by the irradiated and confined liquid-cell vessel alter the system from normal “bulk-like” behavior in multiple ways. These artefactual LCTEM effects will leave their fingerprints in the motion behavior of the diffusing objects, which can be revealed through careful analysis of the object-motion trajectories. Improper treatment of the motion data can lead to erroneous descriptions of the LCTEM system’s conditions. Here, we advance our anomalous diffusion object-motion analysis (ADOMA) method to extract a detailed description of the liquid-cell system conditions during any LCTEM experiment by applying a multistep analysis of the data and treating the *x*/*y* vectors of motion independently and in correlation with each other and with the object’s orientation/angle.

## Introduction

Liquid-cell transmission electron microscopy (LCTEM) is an emerging tool for studying solvated nanostructures.^1,2^ This holds promise for examining how these structures form, and undergo transformations, including through multicomponent reactions and thermally driven processes.^3−9^ One particular area of interest is the *in situ* imaging of nanomaterials in motion, during materials chemistry processes, which can directly reveal the underlying mechanisms and step-wise kinetics in the system observed. However, development of both experimental and data analysis tools is needed to enable true particle tracking. At the core of this is the fact that to form an image by LCTEM, the beam necessarily should irradiate the sample in a manner that can influence the behavior of the sample. The presence of artefactual observation/experimental effects is not unique to LCTEM and is present in some form in all direct imaging/videography techniques, such as in situ atomic force microscopy (cantilever-probe artefacts) or in situ fluorescence microscopy (laser artefacts). In LCTEM specifically, the incident electrons are scattered by the enclosing windows, the liquid itself, and the sample within the liquid. These electron–sample interactions (primarily elastic scattering) give rise to the desired LCTEM data, signal, and contrast in images and videos, while simultaneously, the electron beam causes a number of detrimental effects including (a) radiolysis/ionization reactions of solution, (b) chemical modifications of solvated nanostructures and windows, (c) charging of window and nanomaterial, (d) knock-on-damage of inorganic structures, and (e) nucleation, aggregation, or other transitions related to secondary (or higher order) reactions that result from radiolysis.^1,2,10,11^ LCTEM data are always plagued by electron-beam effects and artifacts that alter the system being observed, which one generally attempts to reduce by using low flux conditions. However, the magnitude of the effects within the irradiated liquid-cell vessel that alter a system’s dynamic behavior is very difficult to directly probe experimentally during a LCTEM experiment. In LCTEM videography, subdiffusive motion consisting of temporary trapping and intermittent walks or flights have been found in the motion of solvated nanostructures diffusing at or near the LCTEM windows in both organic and inorganic nanoparticle (NP) systems.^5,10^ Such NP motion behavior, which is anomalous, altered by artefacts imposed by the experimental system, has been attributed to secondary charging effects, whereby the incident beam induces positive window charging, which generates an electric field within the liquid cell (for insulating windows, e.g., Si3Nx).^11^ These occur at the windows and/or on the NPs themselves, caused by the irradiating electron beam,^5,10,12−14^ and/or to changes in the solution pH/chemistry or the NP or window surface chemistry from e-beam radiolysis reactions.^15,16^ Recent studies also suggest the presence of spatially-varying electric fields generated during LCTEM observation, which could have an influence on NP-window interactions and, hence, NP diffusion.^11^ These experiment-specific artefactual phenomena collectively affect the diffusion of nanoscale objects observed by LCTEM, which we aim to better understand through the analysis and treatment of experimental LCTEM data.

**Figure 1:**
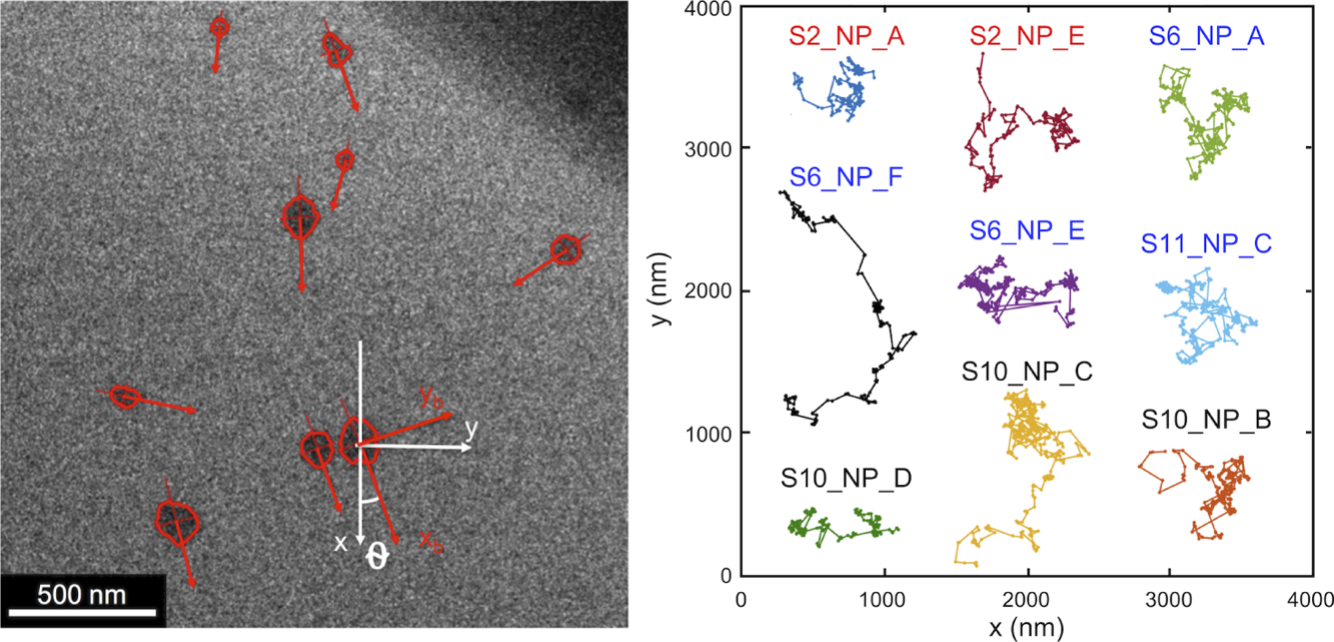
Frame from one LCTEM video of micelles in solution.^5^ The shapes of the micelles are not spherical (left). The rotational motion of the micelles is monitored by the angle formed by the main axis of the ellipse and the cell axis (lab frame). The coordinates in both systems, white axes for lab, and red for body frame, are shown. Several of the extracted LCTEM micelle trajectories are depicted on the right (label color indicated LCTEM flux; black labels for 1.6 e^–^/Å^2^ s, blue labels for 2.6e^–^/Å^2^ s, and red labels for 5.6 e^–^/Å^2^ s.

Herein, we make use of our anomalous diffusion object motion analysis (ADOMA), which is a universal method of analyzing real life-hierarchical distributed time series of a measured quantity. It can be trajectories of NPs in LCTEM experiments,^5,10^ of lipids in bilayer systems,^17^ of fullerenes on gold surfaces,^18^ of microsaccadic movements in eye-head control experiments,^19^ of temperature and conductivity fluctuations of outgassing ions just over the crater of submarine volcanoes,^20,21^ and others. Common in all these diverse scientific fields is the stochastic character of the quantity under study, whose nature can be successfully unveiled by ADOMA. We demonstrate it is now possible to correlate anisotropies in the directional and rotational components of motion to generate a semiempirical description of the forces within the LCTEM experimental system that are driving motion. The diffusion trajectories of NPs in solution can be considered as the manifestation of a stochastic process; see Figure 1 (right).

Their systematic analysis provides information on the nature of the diffusional motion and the system or environment in which that motion occurred.^5,10,14,15^ In previous work, we analyzed lateral motion of elliptical (ca. 0.7 aspect ratio) polymeric micelles in water (with low buffer conc.) from LCTEM videography data, a frame of them is depicted in Figure 1 (left).^5,10^ The results showed that in the majority of cases, across flux used, ca. 1.6–5.6 e^–^/Å^2^ s, micelle motion was subdiffusional, with very few superdiffusional exceptions.^5,10^ Micelle motion often showed a multifractal character, the result of occasional trapping periods when the micelle is pinned at the window surface between active periods of motion. Some of the micelles also exhibit periods of motion with monofractal nature, specifically fractional Brownian motion (fBm), driven by fractional Gaussian noise (fGn); see for definitions of fBm and fGn the seminal work of Mandelbrot.^22^ The LCTEM flux was found to affect micelle motion, albeit in a complex manner. At higher flux, micelles experience fewer and shorter trapping events reflected on a smoother intermittent structure of walks, while the character of subdiffusive motion between walks was largely unaffected by flux. Many questions remain as to how the collective conditions of an irradiated liquid-cell vessel influence dynamic behaviors, which we address through a new treatment of the micelle motion trajectories.

LCTEM micelle motion captured using TEM cameras occurs at or near the membrane surface, and the micelles experience a landscape of potential maxima (barriers) and minima (binding sites), whose effects will be unique for each micelle because of the amorphous and dynamic arrangement of chains within micellar assemblies. If a single type of binding site were present between the micelle and window, then, the distribution of trapping times, ψ(*t*), would decrease exponentially. If instead, there were a broad spectrum of binding sites, the waiting times would follow a power law distribution, which has been reported for polymers at solid–liquid interfaces.^23^ Motion analysis of molecular dynamics simulations has provided evidence for the existence of nonequilibrium structures at polymer–solid interfaces characterized by strong and segment-specific interactions at the surface, and even single PMMA monomers have been found to bind to surfaces under long-lived nonequilibrium orientations.^24^ Single-molecule tracking at a solid–liquid interface has shown that molecules undergo intermittent random walks with non Gaussian displacements, and intermittent hopping has been proposed as the mechanism of explaining molecular surface diffusion at a solid–liquid interface.^25^ Generally, power law distributions of both waiting times and length steps are specific to the dynamics of strongly adsorbed systems over certain time and length scales,^26^ that is, polymer unimer/micelle motion at a solid window is expected to be anomalous generally but might be additionally affected by factors specific to the LCTEM experimental system related to the irradiation of the TEM beam. Understanding these additional artefactual effects on any system under observation by LCTEM videography is critical to properly interpret the LCTEM results. Here, we report a complete video data analysis methodology to extract a semiempirical description of the energy landscape within the experimental system in which the motion occurred, which we apply to understand how electron-beam irradiation affects the liquid-cell vessel consisting of silicon nitride windows and an aqueous polymer micelle solution; this methodology is applicable beyond LCTEM and can be used to extract a semiempirical description of the system conditions from any experimental video data file that contains observation/experimental artefacts.

**Figure 2:**
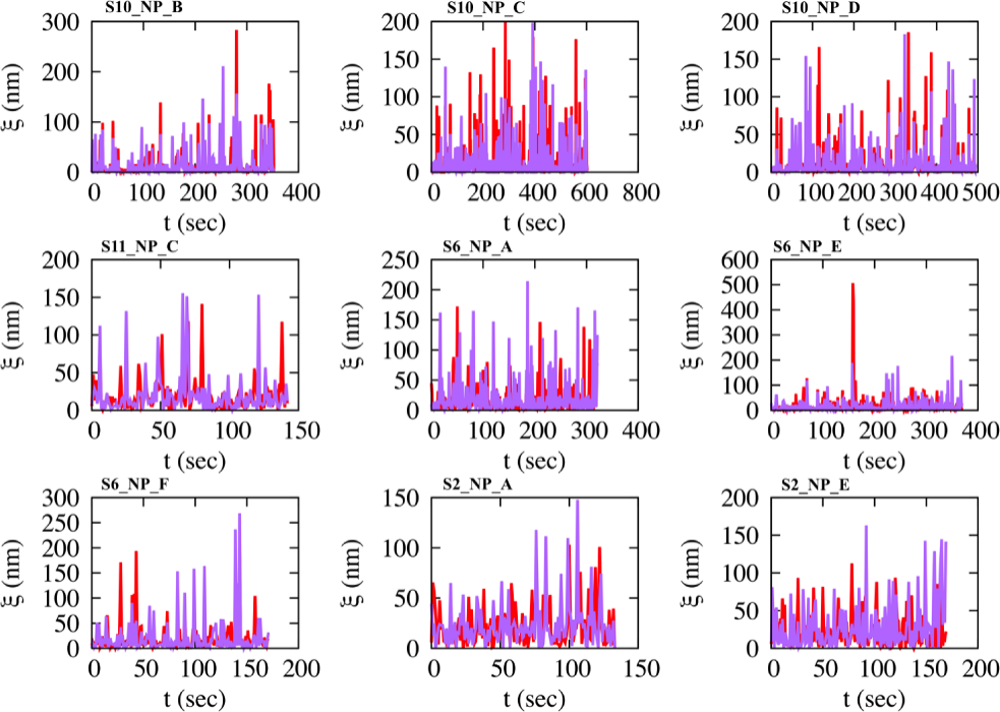
Time series of absolute lengths, ξ, of consecutive movements of micelles at different radiation fluxes show the intermittent structure. Red/purple for movements in the *x* and *y* axes, respectively.

ADOMA has been expanded here to separate analyses of the *x*- and *y*- step and rotational components of the micelle trajectories, determining their cross-correlations. The analysis shows a consistent anisotropy of diffusional motion that exists in {*x*, *y*} axes, and that the mean square displacements and the variances for their two axes and for all micelles do not scale in the same way, Table 1. Micelle motion on each respective axis exhibits similar trends as the overall lateral motion; intermittent walks are interrupted by trapping events, Figure 2, and there is no preferential motion direction over the ensemble of micelles. The directionality of each micelle in motion is unique and appears to be independent. The analysis showed that motion in each axis presents a multifractal character indicated by the specific form of the extracted structure functions, which have convex shapes when plotted as function of the order of the moment.^5,10^ These findings indicate the existence of a complex stochastic process that results from the multiplication/convolution between at least two stochastic processes, which are known to reflect complex environments that favor the appearance of multiple processes evolving at similar time scales in direct competition.^27−29^ Our analysis suggests that the beam effects (i) convolute the natural state of the liquid-cell vessel containing polymer micelles by reducing of the potential energy surface and (ii) alter the electrostatic interactions between micelles and surface, by inducing a bulk positive window charge and/or by weakening local hydrogen bonding between micelles and surface.

**Table 1:** Estimated Scaling Exponents of MD (γ_MD_) and MSD (γ_MSD_) According to eq 3 for *q* = 1 and *q* = 2, Respectively, and of Variance, (γ), According to eq 1^a^

	S10_NP_B		S10_NP_C		S10_NP_D		S11_NP_C		S6_NP_A	
	*x*	*y*	*x*	*y*	*x*	*y*	*x*	*y*	*x*	*y*
γ_MD_	0.494	0.660	0.489	0.558	0.551	0.549	0.652	0.491	0.474	0.589
γ_MSD_	0.712	1.084	0.819	0.979	0.834	0.886	1.155	0.696	0.843	0.993
Γ	0.488	0.891	0.677	0.851	0.552	0.713	0.982	0.380	0.727	0.790
*z*_*x*_*i*_,θ_(*q* = 2)	0.583	0.778	0.501	0.629	0.577	0.574	0.649	0.515	0.447	0.583
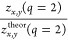	0.855	0.874	0.852	0.858	0.893	0.860	0.905	0.975	1.00	0.86

	S6_NP_E		S6_NP_F		S3_NP_A		S3_NP_E	
	*x*	*y*	*x*	*y*	*x*	*y*	*x*	*y*
γ_MD_	0.495	0.479	0.725	0.730	0.552	0.528	0.558	0.524
γ_MSD_	0.708	0.770	1.185	1.228	1.054	0.969	1.070	0.961
Γ	0.466	0.573	0.958	1.083	0.986	0.846	1.016	0.853
*z*_*x*_*i*_,θ_(*q* = 2)	0.504	0.487	0.753	0.683	0.581	0.640	0.580	0.548
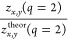	0.787	0.739	1.216	1.206	1.02	0.975	0.988	1.015

a Obtained correlation coefficients for *q* = 2 of translational and rotational movements, namely, *z*_*x*,*y*_(*q* = 2), *z*_*x*,θ_(*q* = 2), *z*_*y*,θ_(*q* = 2). Correlation coefficients have been obtained by use of eq 5.

## Methods

In complex environments, experimental and theoretical studies have shown that the variance, *W*(*t*), of a micelle grows as a power law of time^5,10,17,30−32^
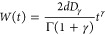
1where *D*_γ_ is a generalized diffusion coefficient in units of *L*^2^*T*^–γ^, *d* is the dimension of the space where the motion evolves, Γ() is the gamma function, and γ is the exponent classifying the motion; subdiffusion for 0 < γ < 1, linear or Brownian under certain circumstances for γ = 1, and superdiffusion for 1 < γ < 2. For discrete data sets, eq 1 can be obtained as the time average, eq 2
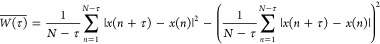
2where *N* is the total number of points of the trajectory, and τ is the time lag taking values up to *N*/10.^5,10,17,30^ In eq 2,  is the mean-squared displacement (MSD) or second moment, and  is the mean displacement (MD) or first moment. MD and MSD may scale similarly to eq 1, with exponents γ_MD_ and γ_MSD_. When γ_MSD_ = 2γ_MD_, then variance and MSD scale with the same exponent, γ, and the process is monofractal—a unique scaling exponent exists. We consider the norm of the displacements, ∥Δ*X*_*i*_(τ)∥ = |*X*_*i*_(*n* + τ) – *X*_*i*_(*n*)|, *n* = 1, 2, ..., *N* – τ, where *X*_*i*_ stands either for *x* or for *y* axes or for θ, where θ is the orientation angle formed by the lab frame and the body frame, Figure 1. Notice that ξ = ∥Δ*X*_*i*_(1)∥ is illustrated in Figure 2. By hypothesis, the moments of order *q* > 0 of the displacement depend only on the time increment τ. We introduce the structure function *z*(*q*) defined as^33−36^
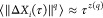
3

The form of *z*(*q*), which is also written as *z*(*q*) = *qH*(*q*) with *H*(*q*) being the generalized Hurst exponent, provides insights into the kind of random motion, see below. If *H*(*q*) is not a linear function of *q*, then multiple scales exist and this property is called intermittency.^34^Eq 3 is a generalization of eq 1, and the scaling exponents γ_MSD_ and γ_MD_ are connected to the value of *z*(*q*) for specific moments, namely, *z*(*q* = 2) = γ_MSD_, *z*(*q* = 1) = γ_MD_. Classically, MSD is obtained and whenever it is *z*(*q* = 2) = 1, the process is classified as Brownian motion. Anomalous diffusion starts when *z*(*q* = 2) ≠ 1.^28,29^ The condition *z*(*q* = 2) = 1 is not strong to characterize the whole process as Brownian;^37^ it is better the characterization be carried out by using the full form of the structure function. Some special forms of the *z*(*q*) provide direct classification of the type of the stochastic process underlying the motion. *H*(*q*) = 0 implies *z*(*q*) = 0, and the process corresponds to a stationary one. If *H*(*q*) = *H*, then, *z*(*q*) = *Hq*, and the process is classified as fractional Brownian Motion (fBm)—monofractal process—and is further characterized as subdiffusive for 0 < *H* < 0.5, Brownian for *H* = 0.5, and superdiffusive for 0.5 < *H* < 1. If *z*(*q*) is a bilinear function of the order of the moment, then if the slope of the second line is higher than that of the first one, then the process corresponds to a Lévy Walk (LW). If instead, the slope of the second line is zero, then motion is classified as Lévy Flights (LFs).^38,39^ Any departure from linearity is a strong indicator against Brownian, fractional Brownian, Lévy, and fractional Lévy models, which all are additive. A convex shape of *z*(*q*) as function of the order of the moment, bending over linearity, underlines multifractality and thus classifies multiplicative processes. Multifractals can be seen as a one-to-one mapping of a monoscaling process to a multiscale one. Such is, for example, a compound fractional Brownian motion, *B*_fBm_(τ), whose time variable, τ, corresponds to the accumulation of a distribution *g*(*t*) from 0 to *t*, τ(*t*) = ∫_0_^*t*^*g*(*t*^′^)d*t*^′^, where *g*(*t*) is an α-stable Lévy distribution with 0 ≤ α ≤ 2.^40^ Multifractals are nonstationary, nonlinear, and nonadditive random processes. Among them, universal multifractals are likely to be ubiquitous, and their structure function reads^41^
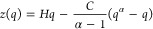
4where *H* is the mean fluctuation exponent, *H* = *z*(*q* = 1), and it plays the role of the Hurst exponent. For *H* = 0, the time series are stationary, while for *H* ≠ 0, the resulted time series correspond to fractional integration of stationary increments. *C* takes only positive values and indicates intermittency; the higher the value of *C*, the stronger the intermittent effects. The Lévy index α indicates the class to which the probability distribution belongs to. It provides information about the relative variation of intermittency around the mean. If α = 0, the structure function describes a monofractal process. For α = 1, the structure function reads *z*(*q*) = *Hq* – *Cq* log(*q*) and the variation of intermittency around the mean draws steps from a Cauchy–Lorentz distribution. For α = 2, a log-normal distribution describes intermittency variations around the mean.^42^ For the estimate of eq 3, we use the following time average^5,10,17,36^
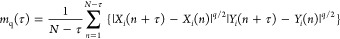
5

Equation 5 provides the moments, *m*_q_(τ), as a function of the elapsed time, for (i) the autocorrelation, which is obtained when *X*_*i*_(*n*) = *Y*_*i*_(*n*). The extracted moments are then fitted to a power law of the form τ^*z*(*q*)^, where the exponent *z*(*q*) is the value of the structure function for the moment *q*. We obtain moments in the range 0.25 ≤ *q* ≤ 3. We estimate the structure functions *z*_*x*_(*q*), *z*_*y*_(*q*), and *z*_θ_(*q*) for the movements in *x* and *y* axes as well for the rotational ones, and for (ii) the cross-correlation of *X*_*i*_(*n*) and *Y*_*i*_(*n*), when *X*_*i*_(*n*) ≠ *Y*_*i*_(*n*).^43,44^ Following the same procedure as above, we estimate the function *z*_*X*_*i*_,*Y*_*i*__(*q*) = *qH*_*X*_*i*_,*Y*_*i*__(*q*), where *H*_*X*_*i*_,*Y*_*i*__(*q*) is the bivariate Hurst exponent. Its value for *q* = 2 can indicate (a) whether the time series are uncorrelated, correlated, or anticorrelated and (b) whether *X*_*i*_(*n*) and *Y*_*i*_(*n*) have the same stochastic mechanism. If *z*_*X*_*i*_,*Y*_*i*__(*q* = 2) is close to 0.5, the time series, *X*_*i*_(*n*) and *Y*_*i*_(*n*), are uncorrelated. They are anticorrelated for a value lower than 0.5 and correlated for a value higher than 0.5. The stochastic processes have the same origin if *z*_*X*_*i*_,*Y*_*i*__(*q*) ≈ {*z*_*X*_*i*__(*q*) + *z*_*Y*_*i*__(*q*)}/2.^43,44^

## Results and Discussion

LCTEM experiments involving solvated block copolymer micelles in dPBS buffered water (using SiNx windows and 200 keV) were conducted at three electron flux, 1.6 e^–^/Å^2^ s (i.d. S10_NP_B, S10_NP_C, and S10_NP_D), 2.6 e^–^/Å^2^ s (i.d. S11_NP_C, S6_NP_A, S6_NP_E, and S6_NP_F), and 5.6 e^–^/Å^2^ s, (i.d. S2_NP_A, and S2_NP_E).^5^ LCTEM videos of micelle motion were recorded at 1 fps (frame per second), frame exposure time of 0.3 s (ca. 0.7 s dead time), with nanometer spatial resolution, and the spatial coordinates (*x*, *y* trajectories, Figure 1 right) of the micelles were extracted for each micelle using multiobject tracking analysis (MOTA),^45,46^ details are given in Section I of Supporting Information. LCTEM necessarily employs projection [transmission] imaging, where only buoyant trajectories sustained in the plane perpendicular to the beam can be recorded (*x*–*y* diffusion). LCTEM video files are available in the Supporting Information of Parent et al.^5^

Analysis of the raw LCTEM data show that micelle motion for both the *x, y* axes possess intermittent structures of step lengths between consecutive movements (Figure 2). These structures change form for the different radiation fluxes, indicating a flux dependence on micelle LCTEM motion.

**Figure 3:**
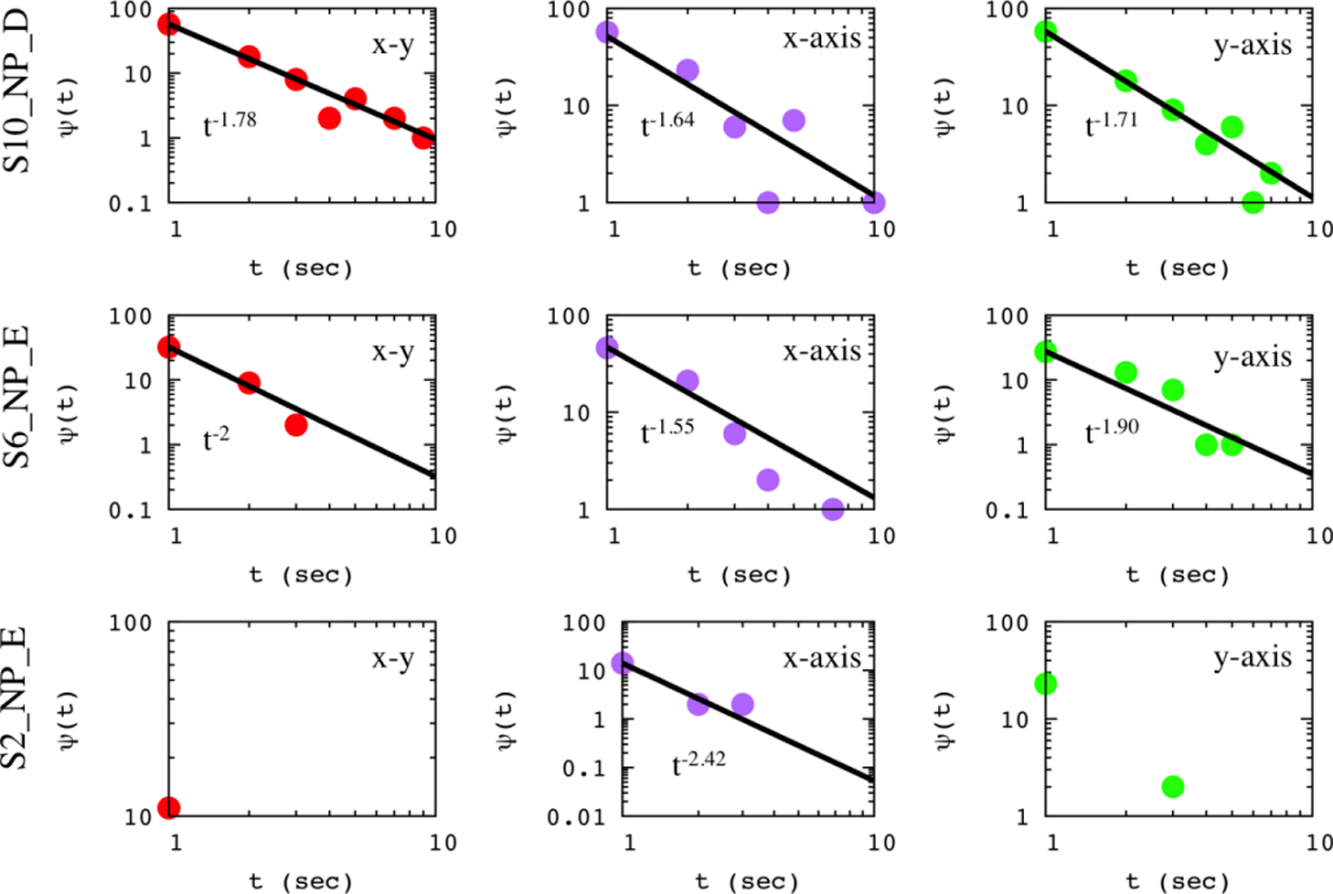
Waiting times distribution ψ(*t*) versus the elapse time *t* for three representative examples. Color code: red for the lateral motion of a micelle, purple for the motion in the *x* axis, and green for the motion in the *y* axis.

For low flux, motion consists of significant periods of complete immobilization (trapping events, which could be associated to multiple binding sites), interrupted by long jumps. Instead, for high flux, micelles are not immobilized, and motion consists of nonvanishing small jumps interrupted by long ones. At the two lower flux examined here (1.6, 2.6 e^–^/Å^2^ s), trapping events are associated with long-tailed distributions of waiting times,ψ(*t*), for all micelles; three representative examples are depicted in Figure 3.

The obtained scaling exponents differentiate the motion in the *x* and *y* axes. Notice that the scaling exponent for lateral motion is different than those describing movements in each perspective axis, a fact that highlights specific-segment type-like interactions between the micelle and the membrane, which must be anisotropic and heterogeneous (zwitterionic) across the window surface at these flux conditions.

**Figure 4:**
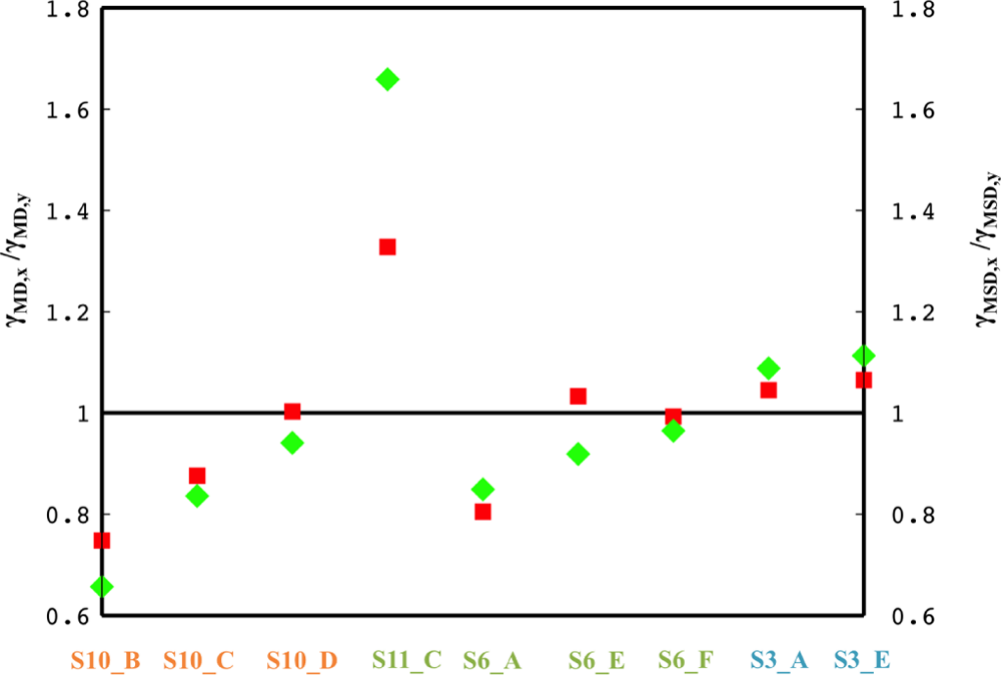
Ratio of the scaling exponents, γ_MD_,_*x*_/γ_MD_,_*y*_ (red squares) and γ_MSD_,_*x*_/γ_MSD_,_*y*_ (green rhombs) is illustrated. The i.d. of each micelle is indicated in the vertical axis of the graph, and the color code stands for the different irradiation flux; orange for 1.6 e^–^/Å^2^ s, dark green for 2.6 e^–^/Å^2^ s, and light blue for 5.6 e^–^/Å^2^ s.

Independent of the radiation flux, the second moment (MSD) and the variance of the motion in both *x* and *y* axes do not scale with the same exponent for the majority of the micelles (Figure 4, Table 1) further indicating anomalous character of the motion.

We note that normal diffusion (Brownian motion) in the case of raw data recorded by experimental imaging techniques, which contain noise and blur because of imperfect optics and cameras, might appear to be anomalous. In such cases, the origin of this discrepancy is either because of static localization error or dynamical error (blur motion).^47−49^ This scenario has been extensively considered here by using well-established methods^47−51^ and has been discarded as origin of the anomalous motion; see detailed analysis and discussion at Sections II and III of Supporting Information.

Further analysis of the raw LCTEM data, see Section IV of Supporting Information for details, delivered two key findings: (i) micelle motion does have anomalous character; for all micelles, the relation γ_MSD_ ≠ 2γ_MD_ holds true for movements in the *x* and *y* axes, and (ii) for low radiation flux, trapping events are associated with power law distributions of waiting times, Figure 3, while immobilization becomes rather a rare event for higher irradiation flux. Trapping events can be an indication of continuous time random walk,^52^ which is a nonergodic process and is associated to ageing effects, which is a characteristic property of nonstationary stochastic processes.^28,53,54^ Ageing is not observed in the data here likely because of the energy (window charging, generating *E*-field) continuously provided by the sustained electron beam, which reduces the number of trapping events as a function of its strength (e.g., flux); however, when the time series are reversed, ageing effects clearly appear; see Section IV of Supporting Information. Time reversal of the series starts from a situation, *t*_final_, where the effect of the e-beam has been continuously summed and goes toward a situation, *t*_initial_, where the same effect is constantly decreased. The accumulated energy delivered by the beam reduces the number of trapping events, an action likely connected with the effect of the beam on the windows. Window charging of insulating LCTEM Si3Nx windows by the electron beam is largely uniform across irradiated area that is within the field of view of the TEM video camera,^11^ generating a positive potential that can begin to overpower the effective pinning strength of local surface traps with increasing flux. This argument is strengthened by the fact that for the highest flux used here, power law distributions of waiting times have not been found. Figure 3 indicates the influence of flux on window–micelle interactions, which has been previously observed.^10^

Figure 4 shows the ratio γ_MD_,_*x*_/γ_MD_,_*y*_ and γ_MSD_,_*x*_/γ_MSD_,_*y*_ for movements in the *x* and *y* axes. The value of 1 for these ratios indicates that movements in *x* and *y* axes scale in the same way. When both ratios take values close to 1 but the relation γ_MSD_ ≠ 2γ_MD_ does not hold true, then the ratio of the corresponding scaling exponents for the variance is different than one, as is the case for the micelles with i.d. S10_NP_D and S6_NP_F. Such complex behavior, apart from the anomalous character of the motion, underlines the necessity of using the full range of moments in characterizing the type of motion. Furthermore, for all micelles, the extracted values of these ratios highlight the existence of a consistent anisotropy in motion in *x* and *y* axes.

**Figure 5:**
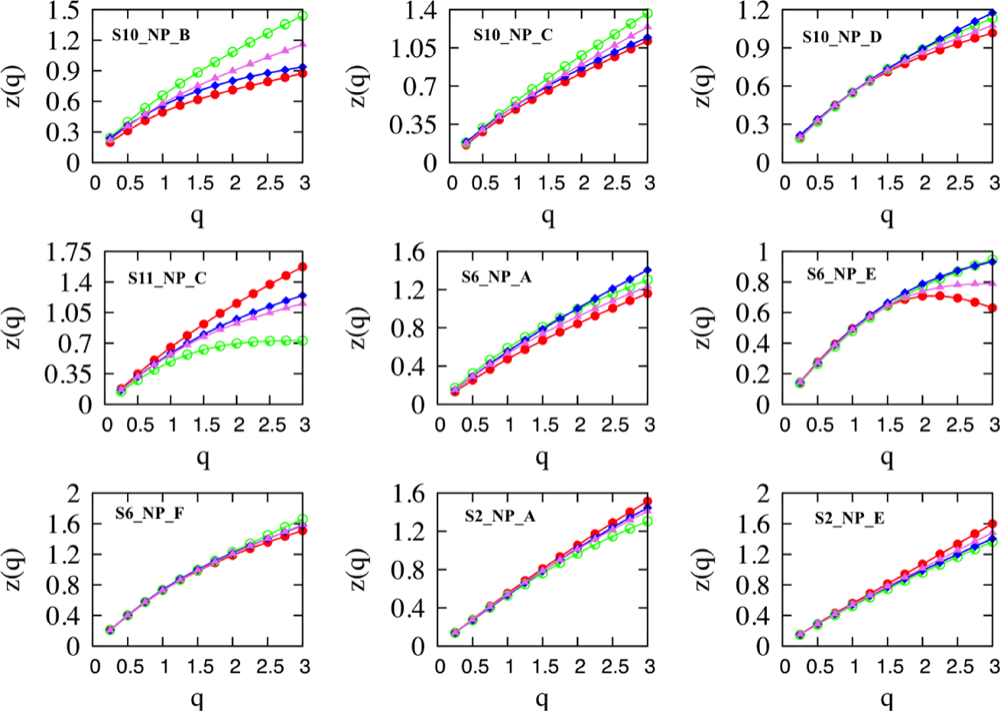
Structure functions for motion in *x*, *y* axes; red for *z*_*x*,*x*_, and green for *z*_*y*,*y*_. In blue, the cross-correlation of movements in *x* and *y* axes. In violet, the theoretical value of the cross-correlation *z*_*x*,*y*_^theor^(*q*) = (*z*_*x*,*x*_(*q*) + *z*_*y*,*y*_(*q*))/2. Notice that *z*_θ,θ_(*q*) is not illustrated because it takes zero values for all *q*’s.

Figure 5 shows *z*_*x*,*x*_(*q*), *z*_*y*,*y*_(*q*), *z*_*x*,*y*_(*q*), and *z*_*x*,*y*_^theor^(*q*), which are the estimated structure functions for micellar motion, calculated via eqs 3–5. The obtained structure function for rotational motion indicates stationary process for all micelles, *z*_θ,θ_(*q*) ≈ 0 for all *q*’s, and are not drawn. Each structure function has been fitted by eq 4, and the obtained parameters *H*, *C*, and α are listed in Table 2, where the structure functions for cross-correlations between translational and rotational movements, *z*_*x*,θ_(*q*) and *z*_*y*,θ_(*q*), are also listed. All structure functions have convex shapes as functions of the order of the moment, except *z*_*x*,*x*_(*q*) and *z*_*x*,θ_(*q*) for micelle S6_NP_E. The convex shape of structure function confirms the multifractal character of micelles’ motion. Furthermore, the estimated structure functions differ for motion across the *x* and *y* axes, reaffirming the existence of persistent anisotropy.

**Table 2:** Analytical Forms of all Obtained Structure Functions are Provided^a^

	S10_NP_B	S10_NP_C	S10_NP_D	S11_NP_C	S6_NP_A
*Z*_*x*,*x*_(*q*)	0.489*q* – 0.184*q* log(*q*)	0.486*q* – 0.106*q* log(*q*)	0.549*q* – 0.192*q* log(*q*)	0.644*q* – 0.062 (*q*^2^ – *q*)	0.473*q* – 0.077*q* log(*q*)
*Z*_*y*,*y*_(*q*)	0.660*q* – 0.167*q* log(*q*)	0.558*q* – 0.095*q* log(*q*)	0.550*q* – 0.156*q* log(*q*)	0.476*q* – 0.121 (*q*^2^ – *q*)	0.587*q* – 0.135*q* log(*q*)
*Z*_*x*,*y*_(*q*)	0.562*q* – 0.229*q* log(*q*)	0.523*q* – 0.129*q* log(*q*)	0.554*q* – 0.151*q* log(*q*)	0.575*q* – 0.082 (*q*^2^ – *q*)	0.551*q* – 0.074*q* log(*q*)
*Z*_*x*,θ_(*q*)	0.397*q* – 0.145*q* log(*q*)	0.318*q* – 0.095*q* log(*q*)	0.358*q* – 0.102*q* log(*q*)	0.352*q* – 0.027 (*q*^2^ – *q*)	0.252*q* – 0.043*q* log(*q*)
*Z*_*y*,θ_(*q*)	0.486*q* – 0.132*q* log(*q*)	0.362*q* – 0.065*q* log(*q*)	0.340*q* – 0.074*q* log(*q*)	0.289*q* – 0.032 (*q*^2^ – *q*)	0.325*q* – 0.032 (*q*^2^ – *q*)

	S6_NP_E	S6_NP_F	S3_NP_A	S3_NP_E
*Z*_*x*,*x*_(*q*)	0.497*q* – 0.144 (*q*^2^ – *q*)	0.708*q* – 0.107 (*q*^2^ – *q*)	0.551*q* – 0.024 (*q*^2^ – *q*)	0.536*q*
*Z*_*y*,*y*_(*q*)	0.470*q* – 0.080 (*q*^2^ – *q*)	0.722*q* – 0.154*q* log(*q*)	0.530*q* – 0.047 (*q*^2^ – *q*)	0.523*q* – 0.062*q* log(*q*)
*Z*_*x*,*y*_(*q*)	0.487*q* – 0.090 (*q*^2^ – *q*)	0.719*q* – 0.101 (*q*^2^ – *q*)	0.536*q* – 0.027 (*q*^2^ – *q*)	0.539*q* – 0.065 (*q*^2^ – *q*)
*Z*_*x*,θ_(*q*)	0.290*q* – 0.038 (*q*^2^ – *q*)	0.416*q* – 0.039 (*q*^2^ – *q*)	0.332*q* – 0.060*q* log(*q*)	0.288*q*
*Z*_*y*,θ_(*q*)	0.276*q* – 0.031 (*q*^2^ – *q*)	0.398*q* – 0.054*q* log(*q*)	0.343*q* – 0.032*q* log(*q*)	0.311*q* – 0.035 (*q*^2^ – *q*)

a For α = 1, the structure function has the form *z*(*q*) = *Hq* – *Cq* log(*q*), for α = 0 *z*(*q*) = *Hq*, and for α = 2 *z*(*q*) = *Hq* – *C*(*q*^2^ – *q*).

In addition to surface chemistry, the shape and size of a nano-object can also influence its diffusional motion,^55^ which can become increasingly influential in spatially confined or anisotropic environments, such as the liquid-cell enclosure under e-beam irradiation. In Figure 1 (left), micelles are marked with red rings (by MOTA algorithm), their major and minor axes are indicated by red vectors (body frame), *x* and *y* coordinates in the lab frame are shown by white vectors, and the orientation angle between the two frames is given by the angle θ. In an isotropic liquid phase, the (local) anisotropy in the diffusion of a nano-object is usually averaged out on a rapid timescale that ranges from the picosecond to the milliseconds, depending on the size of the object. A rough estimate of the time needed for a full rotation, τ_rot_, can be made through the rotational diffusion coefficient *D*^r^ = (*k*_B_*T*)/(8πη*R*^3^) = 1/τ_rot_, where η is the media viscosity, *R* is the particle radius, *T* is the absolute temperature, and *k*_B_ is the Boltzmann constant. The time for a full rotation of a [nanoscale] micelle is in the sub-milliseconds range.^56^ The viscosity of the solution in liquid-cell experiments can potentially increase with decreasing cell thickness, though this effect is not well understood.^12,57,58^ Local window inhomogeneities, in the form of surface defects and different surface moieties, can create interactions that increase the time required for averaging.^5,10,59−61^

Analysis finds a substantial degree of persistent anisotropy in the motion for all micelles that is the propensity of a micelle to move more easily in one direction (*x* or *y*) than the other (scaling exponents of variance listed in Table 1). The motion anisotropy persists for lag times up to ca. ∼ 30 s or longer. Motion anisotropy at such a protracted time scale in a bulk fluid (not confined or irradiated) would require a solution medium with viscosity of 30 Pa s, 4 orders of magnitude greater than that of [lightly buffered] water at room temperature, a viscosity value that is clearly erroneous. This finding highlights the deviation from bulk-like motion of nano-objects in LCTEM experiments, and the danger in treating the data with the assumption that bulk conditions apply; interpretation will be unsound.^62,63^

Rotations, changes in the angle θ in time, are described as a sequence of stochastic events whose positional values are restricted in the range [0:180] degrees for elliptical objects with mirror symmetry. A full 360° rotation of any micelle is never observed during the timescale of all the LCTEM experiments at all flux used. The analysis, by means of application of eq 5, shows that all rotational motions are weakly or strongly stationary-each micelle’s elliptical orientation remains largely fixed during *x*/*y* motion (the calculated scaling exponents are zero or close to zero). Some representative examples are shown at Section V of Supporting Information for individual micelles. The cross-correlation analysis of rotations with the translational motions returns values of *z*(*x*_*i*_,θ)(*q* = 2), *x*_*i*_ = *x*, *y*, within the range 0.447–0.778. A value of *z*(*x*_*i*_,θ) (*q* = 2) ≈ 0.5 defines uncorrelated rotational and translational motions. This relation is not strict for short time series, and time series can be considered as uncorrelated for values lying within a broader range, let say 0.45 ≤ *z*(*x*_*i*_,θ) (*q* = 2) ≤ 0.55. The obtained cross-correlation coefficients *z*(*x*_*i*_,θ) (*q* = 2) mark two trends: (i) uncorrelated rotational and translational motions are found either on both axes or on one of them, or (ii) translational and rotational motion are correlated on both axes. There is not a single case in which rotations are correlated with translations in one axis and anticorrelated in the second one. The latter is in line with the appearance of strong correlation between motions in the *x* and *y* axes; see values of *z*(*x*,*y*)(*q* = 2) in Table 1. For micelle S6_NP_E, the cross-correlation coefficient indicates uncorrelated rotational and translational movements in both axes. For micelle S10_NP_D, rotations have the same slightly correlated dependence with translational motions in both axes. For a number of micelles (S10_NP_C, S11_NP_C, S6_NP_D), rotations are uncorrelated with translations in one axis and correlated with translations in the second one. For the rest of the micelles, rotations are correlated with translations in both axes, but correlations are stronger in one of them. Micelles move faster across that axis, creating the higher correlation with rotational movements. However, the faster axis is usually identified as the minor (*y*) axis of the elliptical structure of the micelle. Exception is micelle S3_NP_A, where the structure function (Table 2) says that the intermittency parameter *C* in *y* axis is almost the double of that of *x* axis. The linear terms of the structure function, 0.551 and 0.530 for *x* and *y,* respectively, designate processes driven by slightly persistent fractional Gaussian noises (fGn). This effect in the absence of any intermittency would deliver almost Brownian motions with scaling exponent 1.10/1.06. The rising of the anomalous character of the motion and accordingly its differentiation across *x* and *y* axes is because of intermittent events caused by the interactions of the micelle with the membrane.

The relation *z*_*x*,*y*_(*q* = 2) ≈ *z*_*x*,*y*_^theor^(*q* = 2) = {*z*_*x*_(*q* = 2) + *z*_*y*_(*q* = 2)}/2 holds for all trajectories and for *q* ≤ 1.5. For some trajectories, there exist deviations for higher order moments (*q* ≥ 1.5), indicating that the differentiation of motion in *x* and *y* axes is likely result of some large steps undertaken in one of the axes.^33^ The structure function for the cross-correlation of movements in *x* and *y* axes shows also a multifractal structure. Note that for all micelles, S6_NP_F is an exception, the linear term of the structure function is close to 0.5 (Table 2), and reflects a cross-correlation coefficient, *q* = 2, close to 1 (perfect correlation) if the *C* term were zero. Intermittency reduces this value of the coefficient (see Table 1), but it still highlights a highly correlated structure between motion in the *x* and *y* axes.

The higher the intermittency parameter *C*, the slower the motion. For all examined trajectories, a consistent anisotropy with respect to the obtained values of the parameter *C* is observed in both the *x* and *y* axes. The anisotropy imposed by intermittent events is then reflected on the scaling laws across each axis. For the higher flux used here, the intermittent structure is washed out with respect to the lower flux and scaling law anisotropy tends to converge.

Prior analysis of LCTEM data has classified object motion as surface-mediated motion,^5,10,15,16^ micelle–window interactions, compounded by the effects of TEM irradiation of sample, solution, and windows. The forms of the obtained structure functions here for all micelles confirm this argument. The multifractal character of the motion points to multiplicative effects between fGn with α-stable Lévy distributions for 0 < α ≤ 2. Two values of α have been identified in the present study: α = 1, which returns a Cauchy–Lorentz distribution, and α = 2, which returns a log-normal distribution (Kolmogorov). One can partly attribute the origin of the fGn to solution molecules. However, there is not a clear sign how TEM irradiation (radiolysis, and/or window charging, reducing H-bonding, etc.) affects these terms, and consequently, micelle’s motion. For low and intermediate flux, there is evidence (Table 2) of strong anisotropy in the linear terms of the structure functions with respect to motion in *x* and *y* axes, where fGn goes from antipersistent (slow axis) to persistent (fast axis). For the same flux, there are also micelles for which the linear terms are quite similar, indicating either antipersistent or persistent fGn. Assuming the effect of TEM irradiation on each water solution molecule is the same, the resulting variations must be because of interactions between the micelles and the membrane. For high flux, this anisotropy weakens and the corresponding linear terms take values very close to white noise (0.5). LCTEM irradiation conditions do not directly drive the motion of micelles; however, through their secondary effects on the solution/polymer or the cell’s windows, and in conjunction with viscoelastic properties of liquid-cell environment, the irradiation conditions shape the overall multifractal character of motion. E-beam irradiation disturbs the liquid-cell vessel, maintaining it constantly out of equilibrium, thus affecting the length scale of interaction. This, in turn, is the origin of anisotropy and is described by long-range correlations and power law distributions.

An interesting feature of the system investigated here is that the micelles are zwitterionic, structurally amorphous, and are less polarizable than the metal-core NPs often studied in LCTEM experiments.^12,15,16^ The nature of the motion along a given direction is mainly affected by secondary effects of the e-beam irradiation conditions, via the direct reduction of the potential energy surface, caused by bulk positive window charging, or by the breaking of chemical/hydrogen bonds and altering electrostatic surface interactions. Unconstrained rotational motion would wash out any motion anisotropy in a submicrosecond time domain, leading to isotropic motion.^56^ Instead, anisotropy persists for at least ∼30 s and rotational motion itself is either uncorrelated or correlated approximately by the same amount in both axes, indicating constrained rotational motion. We also note that intrinsic anisotropy in micelles’ shape likely affects motion’s anisotropy. Assuming the membrane has a roughly homogeneous distribution of pinning sites, it is anticipated that a larger number of binding sites will exist across the longer axis, leading to higher intermittent structures. For low and intermediate flux, the abovementioned argument is in line with the obtained scaling exponents; motion is highly anistropic, suggesting the dominance of the binding sites in directing motion. At the higher flux, however, we find that micelle binding behavior is largely the same for both axes, indicating that the beam has begun to significantly alter the pinning sites and reduce their effective strengths.

## Conclusions

In summary, diffusional motion in an LCTEM experimental system was investigated using multifractal analysis (ADOMA) of video data to extract semiempirical descriptions of the energy landscape during in situ LCTEM observation. The results indicate that imaging conditions, even at the low flux used here, alter the kinetics of nonspherical nano-objects. This is the result of secondary beam effects. We find that micelles in LCTEM exhibit intermittent jumps throughout their motion trajectories, which become less frequent at increasing flux (smoother motion). Intermittent jumps are responsible for establishing motion anisotropy in both axes. The obtained structure functions call for intermittent events drawing steps from an α-stable Lévy distribution, whose time variable is the operational time of environmental fractional Gaussian noise. The motion analysis methodology that we describe provides a more complete understanding of the nature of the dynamical processes observed by LCTEM. Similar shape-anisotropy effects on motion are likely to be present across materials systems at the nanoscale and not only in polymeric micelles. The mathematic treatment that we have introduced has been shown to be effective in extracting insights into the origins of the [experimental] system-specific motion anomalies and can be applied across systems to the treatment of LCTEM videography data broadly.

## Supporting Information Available

Multiobject tracking algorithm (MOTA), experimental methods for MOTA trajectory error calculations, effects of static and dynamic errors on the estimate of the scaling exponent of a diffusing particle, preliminary analysis of raw data, analyzing rotational movements, and analyzing variations in time of micelle size (PDF)
