# Supplementary material for: Complex Nanoparticle Diffusional Motion in Liquid-Cell Transmission Electron Microscopy
Source: J Phys Chem C Nanomater Interfaces. 2020 Jun 10;124(27):14881–90. doi: 10.1021/acs.jpcc.0c03203 (PMC8023318; doi:10.1021/acs.jpcc.0c03203)
Supplement: Supplementary file 1 — jp0c03203_si_001.pdf [file jp0c03203_si_001.pdf]

Supporting Information

for

Complex Nanoparticle Diffusional Motion in Liquid Cell Transmission Electron Microscopy

Evangelos Bakalis,[*,†] Lucas R. Parent,[‡] Maria Vratsanos,[¶] Chiwoo Park,[§] Nathan C. Gianneschi,[||] and Francesco Zerbetto[*,†]

†Dipartimento di Chimica ”G. Ciamician”, Universita di Bologna, V. F. Selmi 2, 40126 Bologna, Italy.

‡Innovation Partnership Building, the University of Connecticut, Storrs, Connecticut 06269, United States

¶Department of Materials Science Engineering, Northwestern University, Evanston, Illinois 60208, United States

§ Department of Industrial and Manufacturing Engineering, Florida State University, Tallahassee, Florida 32306, United States.

|| Department of Chemistry, Department of Materials Science Engineering, and Department of Biomedical Engineering, Northwestern University, Evanston, Illinois 60208, United States.

E-mail: *evangelos.bakalis2@unibo.it; *francesco.zerbetto@unibo.it

Section I: Multi-Object Tracking Algorithm (MOTA)

An automated multi-object tracking algorithm[1,2] was applied to raw LCTEM videos to identify and track the motions of individual micelles for each video. The automated algorithm first identify potential micelles per each image frame of the video and associate the identified micelles over time frames based on their spatial proximity and geometrical similarities; during the process, most faulty identifications are filtered out, and previously unidentified micelles are additionally identified to improve the accuracy of analysis. The association process provides the motion trajectories of the identified micelles. Let *i* denote the index number for denoting the identity of a micelle, let *t* denote the time index. The main output of the tracking algorithm is the silhouette image of micelle *i* observed at time *t* in the form of a set of all image pixel locations on the interior of the micelle, $\left\{\left(\mathrm{x}_n^{(\mathrm{i,t})}, \mathrm{y}_n^{(\mathrm{i,t})}\right); n = 1, \ldots., N_{it}\right\}$. Let $\mathbf{X}_{\mathrm{it}}$ denote the $N_{it}\times 2$ matrix of the pixel locations. We computed the spatial location and the orientation angle of the micelle by taking the column mean and the first eigenvector of $\mathbf{X}_{\mathrm{it}}$

Section II: Experimental Methods for MOTA Trajectory Error Calculations

We have identified several **Potential Sources of Experimental Error** in the determination/quantification of each micelle's trajectory from the raw bright field LCTEM video data, *i.e.* ***Localization Error***, which must be accounted for (as sigma) in the ADOMA analysis. These contribution to the total Localization Error will propagate into error in the accuracy of MOTA in tracking the in center of mass (x,y) position, major and minor axis diameters, and major axis angle of orientation of each micelle in the LCTEM videos.

Potential Sources of Localization Error:

1. **Center of Mass (x,y) Position Error** (unique for each micelle, dependent on that micelle's motion behavior; *can be anisotropic or isotropic depending on that micelle's motion behavior*) due to micelle motion over the 0.3 sec exposure time that the TEM camera was acquiring signal for each frame. Each video frame is given a time stamp (t = *x* sec) at the instant the camera exposure is complete, however, the signal in the video frame contains signal acquired over the 0.3 s exposure time. This creates the Center of Mass Position Error due to object motion.
2. **Isotropic Blurring** effects in the TEM system and in the TEM data *(all can be simulated by Gaussian blur*s):
    a. **Beam Broadening due to multiple/plural scatting** from the [thick] liquid sample (due to the elastic and inelastic scattered electrons that are collected by CCD)
    b. **Beam Broadening due to chromatic aberrations** in the TEM lenses combined with inelastic scattering from sample (from inelastic scattered electrons collected by CCD and the Cs value of the TEM)
    c. **Image Blurring due to pixel binning** (pixel size) → binning of 4 used in these data, where a 4x4 pixel matrix (16 pixels) are averaged into the signal for 1 large pixel (independent of the sample or scattering of the TEM beam)
    d. **Point Spread Function (PSF) of the Gatan Ultrascan1000 CCD camera** used to acquire the LCTEM video data (unique for each TEM camera, and independent of the sample)
3. The above errors in the TEM video data is then translated into error in the accuracy of the MOTA tracking of each micelle's trajectory (center of mass (x,y) position, major and minor axis diameters, and major axis angle of orientation)

METHODS: **Micelle Center of Mass Position Error** due to motion over 0.3 sec camera exposure time:

1. Use the D_{ax} and D_{ay} (nm^2/s) values that have been extracted from each trajectory (from Refs. [3,4]) to calculate the average x- and y-velocities (V_x and V_y) travelled per frame by each micelle during its trajectory, where V_x = sqrt(D_{ax})*(0.3 sec)

2. The Motion Position Smear full width half maximum (FWHM) is set as half the average velocity due to motion in positive and negative x-direction or y-direction: (FWHM) = [(1/2)*V_x] or [(1/2)*V_y]
3. One standard deviation in position error due to motion "smear" is then taken as:

$$\mathbf{1\sigma} = \frac{(^1/_2) \cdot FWHM}{\sqrt{2\ln(2)}} \cong (\mathbf{0.8493}) \cdot \boldsymbol{FWHM}$$

4. $\mathbf{1\sigma}$ values added to Tables S5-S8 as the Center of Mass Position Error (first to rows of each table)

METHODS: **Isotropic Blurring Contributions from Point Spread Function** of Gatan Ultrascan1000 CCD camera (using Boothroyd *et al.* 2013[3]) and from **Beam Broadening due to multiple/plural scattering**:

1. Extract the data values of the Ultrascan1000 point spread function (PSF) from Figure 4 in Boothroyd *et al.*[5]
   - The Boothroyd[5] PSF was measured using the focused beam method: central spot of diffraction pattern through vacuum (using: 100 mm camera length, small selected arear diffraction SAD aperture, energy filter on) is recorder directly on an Ultrascan1000 CCD camera, where the TEM beam diameter is smaller than CCD pixel size (entire TEM beam is incident on one pixel, but residual signal is transferred into adjacent pixels due the camera's PSF). A line scan is then taken across the diffraction spot to measure the blurring of the signal into adjacent CCD pixels.
2. The raw PSF data from Boothroyd *et al.*[5] is plotted in Matlab (**Figure S1**)
3. Using Matlab → The raw data is fit with Smoothing Spline (0.97 smoothing parameter give the best fit) (**Figure S1**)
4. Using Matlab → The raw data is fit with a **Gaussian** (**Figure S1**) to extract sigma (σ)
   - A = 0.9658 (0.836, 1.096)
   - B = 0.002515 (-0.1862, 0.1912)
   - C = 1.72 (1.453, 1.986)
   - R-squared = 0.9583
   - $G(x) = A \cdot e^{-[\frac{(x-B)^2}{C^2}]}$
   - $\sigma = \frac{C}{\sqrt{2}}$
   - $\mathbf{1\ \sigma = 1.2162}$ **CCD pixels (2048 x 2048)**

5. 2K x 2K CCD pixel size for each LCTEM video is converted to image pixel size (in nm)
    - 5 kx mag (1.6 $e^-/Å^2s$), **S10** → 2k pixel = 2.0513 nm → $\mathbf{1\ \sigma = 2.495}$ **nm**
    - 6.5 kx mag (2.6 $e^-/Å^2s$), **S6/S11** → 2k pixel = 1.5974 nm → $\mathbf{1\ \sigma = 1.943}$ **nm**
    - 9.6 kx mag (5.6 $e^-/Å^2s$), **S10** → 2k pixel = 1.0649 nm → $\mathbf{1\ \sigma = 1.295}$ **nm**
6. A **multiple-scattering [Gaussian] blur of 4 nm was added** to mimic the thick liquid layer of the LCTEM sample and the contribution from Cs aberrations in these data. The amount of Gaussian blur was determined manually and qualitatively by comparison with various video frames from LCTEM videos.
7. The **Isotropic Blurring from Ultrascan1000 Point Spread Function** and the Isotropic **Beam Broadening due to multiple/plural scattering** were then used to create simulated LCTEM video frames (see next section)

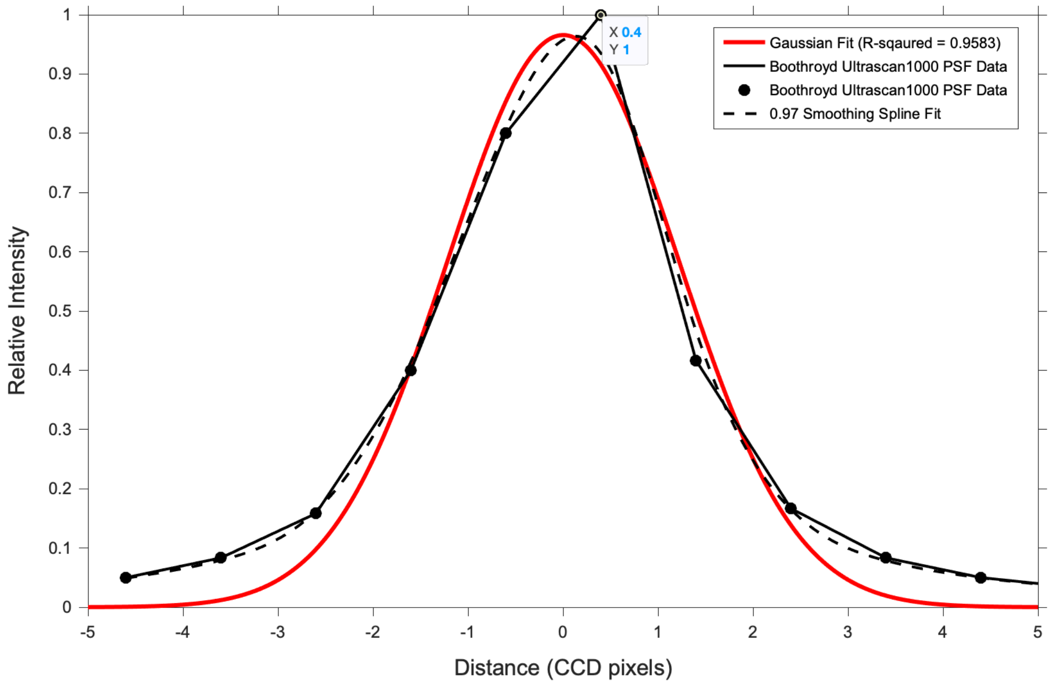

Figure S1. Point Spread Function raw data of Ultrascan1000 CCD camera from Figure 4 of Boothroyd *et al.*[5] (black solid line and black data points). The best Gaussian Fit to the PSF of the Ultrascan1000 CCD is the red curve (R-squared = 0.9583), and the best smoothing spline fit is the dashed black curve (0.97 smoothing parameter).

METHODS: **Simulation of LCTEM Video Frames using the Various Error Contributions**:

1. Combine (sum) the **Micelle Center of Mass Position Error** due to motion with the **Isotropic Blurring from Ultrascan1000 Point Spread Function**, and with the **multiple-scattering [Gaussian] blur of 4 nm**, which gives:

<u>Average video frame Error/Sigma [for all LCTEM data] of *c.a.* 8 nm (8.05 nm)</u>

2. Single frames (0.3 s exposure, 512 x 512) from the raw LCTEM videos from Parent *et al.*[3] (supporting videos S3, S6, S10, and S11) were extracted as 8-bit .tiff images (grey levels: 0 to 255).
3. Using ImageJ → the Average Signal and Noise (σ) of each micelle and of its local background were measured (**Tables S1-S4**) and plotted (**Figure S2**)
4. Using ImageJ → Created a series of 10 simulated LCTEM images for **<u>S3</u>** (**Figure S3**):
    - Create a 2048 x 2048 pixel 8-bit image
    - Set the Background intensity to 102.2
    - Draw a 0.7 aspect ratio (AR) ellipse (100 x 70 pixels) and a 0.8 aspect ratio ellipse (100 x 80 pixels) → Set the Ellipse Intensity to [102.2 – 48.5] = 53.7
    - Rotate the image at: 0°, 15°, 30°, and 45°
    - At 2048 x 2048 → Add Gaussian Blur of 7.51 pixels (**<u>8 nm</u>**)
    - Bin by 4 to render image to 512 x 512 pixels
    - Add the average Background Noise measure from the LCTEM video frames to the simulated 512 x 512 image as σ = 25.8
5. Using ImageJ → Created a series of 10 simulated LCTEM images for **<u>S6</u>** (**Figure S4**):
    - Create a 2048 x 2048 pixel 8-bit image
    - Set the Background intensity to 92.4
    - Draw a 0.7 aspect ratio ellipse (100 x 70 pixels) and a 0.8 aspect ratio ellipse (100 x 80 pixels) → Set the Ellipse Intensity to [92.4 – 50.7] = 41.7
    - Rotate the image at: 0°, 15°, 30°, and 45°
    - At 2048 x 2048 → Add Gaussian Blur of 5.01 pixels (**<u>8 nm</u>**)
    - Bin by 4 to render image to 512 x 512 pixels
    - Add the average Background Noise measure from the LCTEM video frames to the simulated 512 x 512 image as σ = 23.6
6. Using ImageJ → Created a series of 10 simulated LCTEM images for **<u>S10</u>** (**Figure S5**):
    - Create a 2048 x 2048 pixel 8-bit image

- Set the Background intensity to 102.2
- Draw a 0.7 aspect ratio ellipse (100 x 70 pixels) and a 0.8 aspect ratio ellipse (100 x 80 pixels) → Set the Ellipse Intensity to [102.2 – 48.5] = 53.7
- Rotate the image at: 0˚, 15˚, 30˚, and 45˚
- At 2048 x 2048 → Add Gaussian Blur of 7.51 pixels (**8 nm**)
- Bin by 4 to render image to 512 x 512 pixels
- Add the average Background Noise measure from the LCTEM video frames to the simulated 512 x 512 image as $\sigma = 25.8$

7. Using ImageJ → Created a series of 10 simulated LCTEM images for **S11** (**Figure S6**):
    - Create a 2048 x 2048 pixel 8-bit image
    - Set the Background intensity to 118.2
    - Draw a 0.7 aspect ratio ellipse (100 x 70 pixels) and a 0.8 aspect ratio ellipse (100 x 80 pixels) → Set the Ellipse Intensity to [118.2 – 62.8] = 55.4
    - Rotate the image at: 0˚, 15˚, 30˚, and 45˚
    - At 2048 x 2048 → Add Gaussian Blur of 3.90 pixels (**8 nm**)
    - Bin by 4 to render image to 512 x 512 pixels
    - Add the average Background Noise measure from the LCTEM video frames to the simulated 512 x 512 image as $\sigma = 13.7$
8. The major diameters of the 11 micelles in the 4 LCTEM videos (videos S3, S6, S10, and S11) are in the range of c.a. 80 – 170 nm. In each simulated [and reference] video frame, the ellipses drawn cover this range, and only ellipse of this size were MOTA tracked to determine measurement error. Smaller or larger ellipse was excluded in the error determination for not being representative of the micelles' sizes in the experimental LCTEM video data.
9. The collection of simulated LCTEM video frames with blurring added (**Figures S3-S6**) were analyzed using MOTA[3], our automated algorithm for extracting the x,y position, major/minor axis diameters, and angle of orientation of each object in video frames (see next section), and to compare these values to the "reference" video frames (no blurring added) to determine MOTA error in the extraction of the micelles trajectories in the experimental LCTEM videos.

Table S1. Measured values of Micelle Signal (intensity) and Sigma (noise) and local Background Signal and Sigma from single video frames from **LCTEM Video S3** (9.6 kx magnification, 5.6 $e^-/Å^2s$ dose rate, 1.065 nm/pixel at 2048x2048 pixel frame).

**S3**					
**micelle Signal**	**micelle Sigma**	**[micelle - background] Signal**	**background Signal**	**background Sigma (noise)**	**Contrast Noise Ratio**
43.2	18.9	55	98.2	25.1	1.75047421
**52.1**	**18.3**	38.4	**90.5**	**24.8**	**1.24590642**
42.5	20.2	50.6	93.1	24.9	1.57813226
49.8	20.9	47.6	97.4	25.8	1.43359838
71.7	21.3	42.3	114	26.4	1.24700658
59.8	23.5	57.6	117.4	27.5	1.59233932
57.1	21.8	47.9	105	25.8	1.41812832
average		*average*	*average*	*average*	*average*
53.74285714		48.48571429	102.2285714	25.75714286	1.46651221

Table S2. Measured values of Micelle Signal (intensity) and Sigma (noise) and local Background Signal and Sigma from single video frames from **LCTEM Video S6** (6.5 kx magnification, 2.6 $e^-/Å^2s$ dose rate, 1.5974 nm/pixel at 2048x2048 pixel frame).

**S6**					
**micelle Signal**	**micelle Sigma**	**[micelle - background] Signal**	**background Signal**	**background Sigma (noise)**	**Contrast Noise Ratio**
23.6	11.2	41.9	65.5	20.1	1.82096478
34.6	16.6	51.665	86.265	24	1.77047096
46.7	15.9	54.3	101	25	1.83273455
**46.2**	**19.7**	40	**86.2**	**22.6**	**1.334186**
37.5	18.1	47.2	84.7	22.7	1.62575078
42.1	20.6	49.8	91.9	24.2	1.56699877
47.8	16.4	55.5	103.3	24	1.90930323
55.6	16.2	65.1	120.7	26	2.12509198
average		*average*	*average*	*average*	*average*
41.7625		50.683125	92.445625	23.575	1.74818763

Table S3. Measured values of Micelle Signal (intensity) and Sigma (noise) and local Background Signal and Sigma from single video frames from **LCTEM Video S10** (5 kx magnification, 1.6 $e^{-}/Å^{2}s$ dose rate, 2.0513 nm/pixel at 2048x2048 pixel frame).

**S10**					
**micelle Signal**	**micelle Sigma**	**[micelle - background] Signal**	**background Signal**	**background Sigma (noise)**	**Contrast Noise Ratio**
27.9	5.3	35.7	63.6	7.7	3.81911025
**38**	**6.1**	38.1	**76.1**	**10.7**	**3.09337225**
46.3	7.3	57.6	103.9	15.5	3.36193133
56	11.5	70.4	126.4	13.9	3.90232871
82.3	12.4	77.8	160.1	15.8	3.87357215
81.7	13	97.2	178.9	18.6	4.2833101
average		*average*	*average*	*average*	*average*
55.36666667		62.8	118.1666667	13.7	3.7222708

Table S4. Measured values of Micelle Signal (intensity) and Sigma (noise) and local Background Signal and Sigma from single video frames from **LCTEM Video S11** (6.5 kx magnification, 2.6 $e^{-}/Å^{2}s$ dose rate, 1.5974 nm/pixel at 2048x2048 pixel frame).

**S11**					
**micelle Signal**	**micelle Sigma**	**[micelle - background] Signal**	**background Signal**	**background Sigma (noise)**	**Contrast Noise Ratio**
67.5	21	53.1	120.6	26.9	1.55598091
49.6	19.3	64.9	114.5	27.1	1.95070009
60.4	17.3	42.4	102.8	25.7	1.36861094
52.8	19.6	48.6	101.4	25.9	1.49629152
38.5	17.6	37.2	75.7	22.6	1.29866864
**39.5**	**19.5**	32.4	**71.9**	**22**	**1.10211095**
34.8	17.2	43.7	78.5	22.8	1.53010604
average		*average*	*average*	*average*	*average*
49.01428571		46.04285714	95.05714286	24.71428571	1.4717813

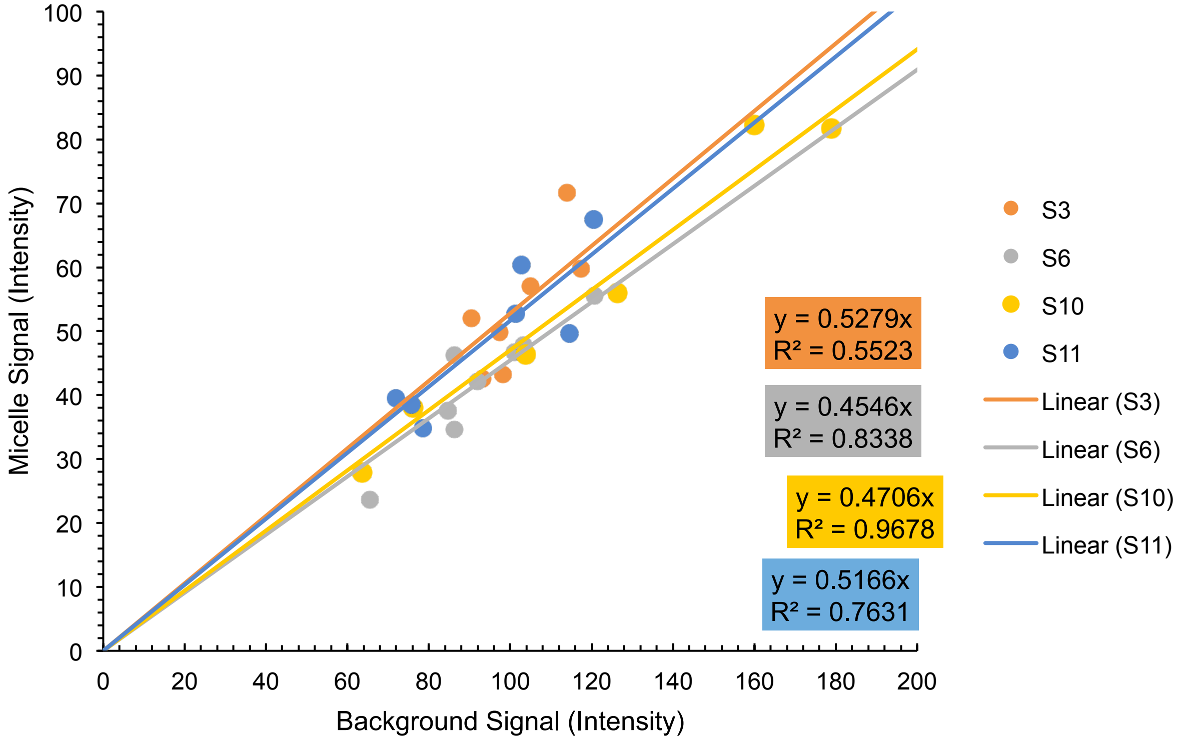

Figure S2. Plot of mean Micelle Signal vs the corresponding local Background Signal extracted from the frames of the LCTEM videos (S3, S6, S10, S11). Individual linear fits are plotted for the data of each of the four videos.

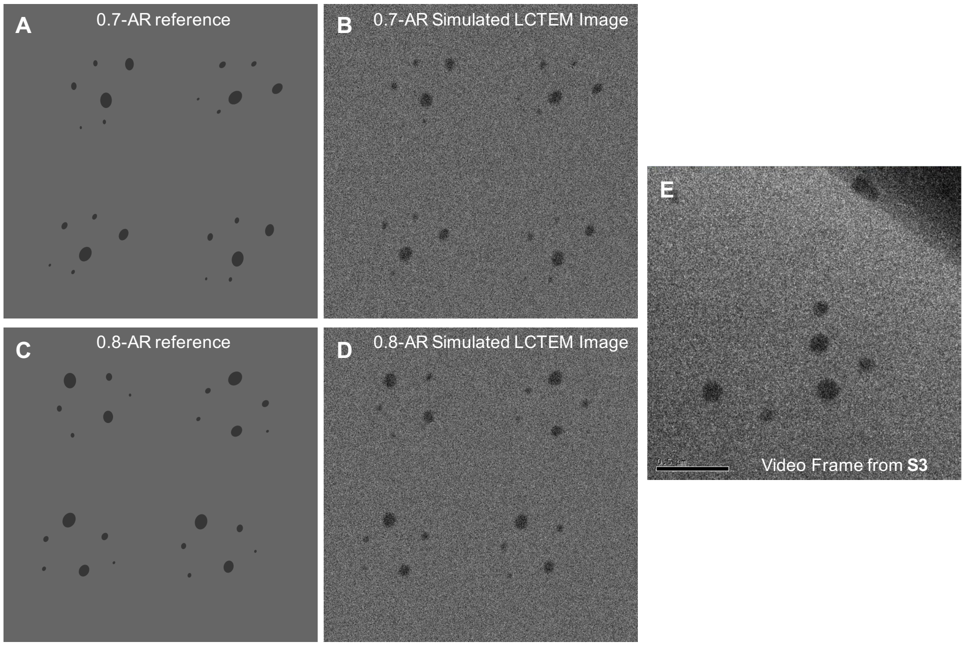

Figure S3. Sample of the simulated images created for video S3. (A) the 2048 x 2048 reference image with 0.7 AR ellipses. (B) One of the 10 simulated 0.7 AR images (512 x512), which is the reference image from A with the simulated LCTEM noise (8 nm Gaussian blur) added. (C) the 2048 x 2048 reference image with 0.8 AR ellipses. (D) One of the 10 simulated 0.8 AR images (512 x512), which is the reference image from C with the simulated LCTEM noise (8 nm Gaussian blur) added. (E) One frame from the raw **S3** LCTEM video for comparison.

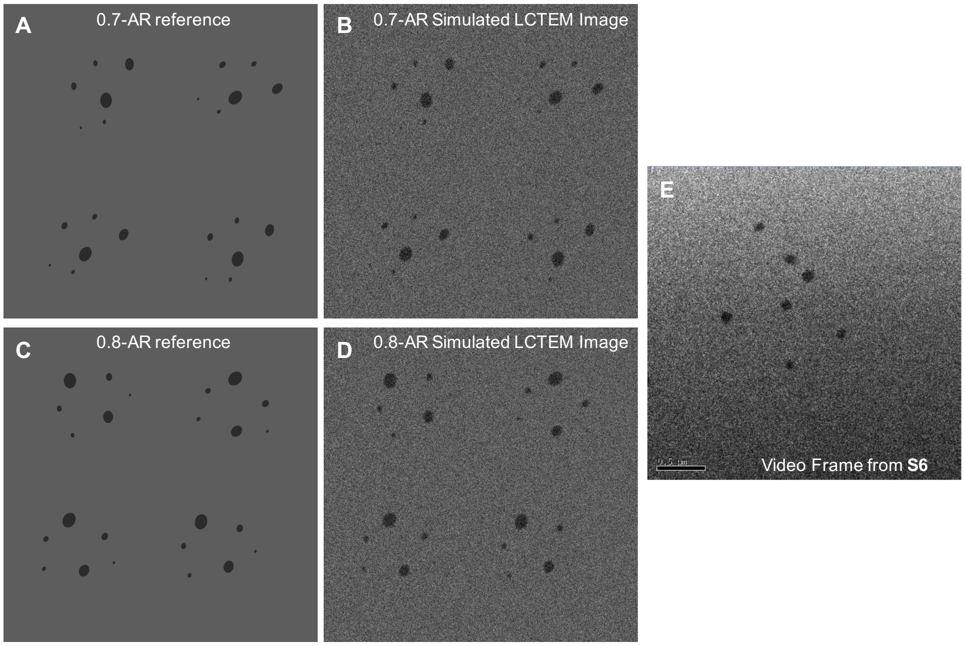

Figure S4. Sample of the simulated images created for video S3. (A) the 2048 x 2048 reference image with 0.7 AR ellipses. (B) One of the 10 simulated 0.7 AR images (512 x512), which is the reference image from A with the simulated LCTEM noise (8 nm Gaussian blur) added. (C) the 2048 x 2048 reference image with 0.8 AR ellipses. (D) One of the 10 simulated 0.8 AR images (512 x512), which is the reference image from C with the simulated LCTEM noise (8 nm Gaussian blur) added. (E) One frame from the raw S6 LCTEM video for comparison.

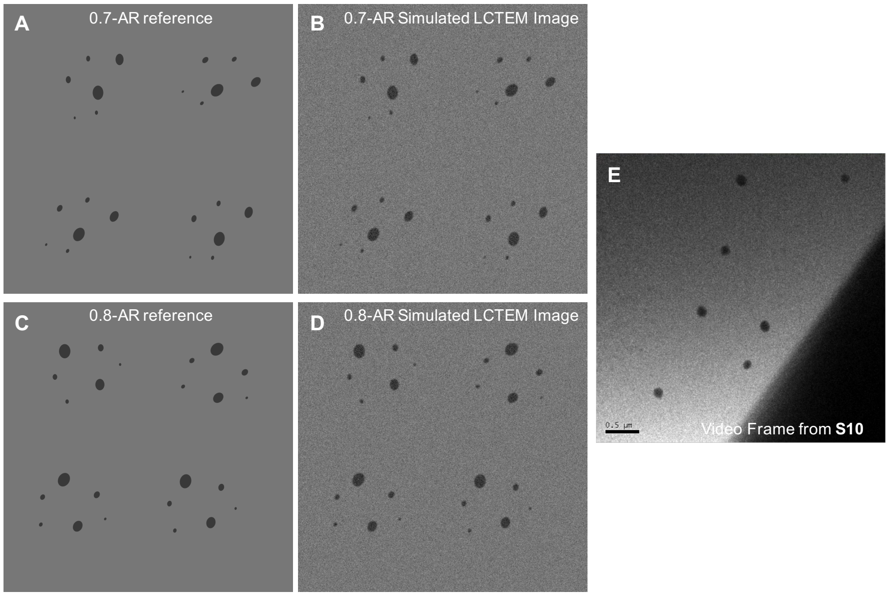

Figure S5. Sample of the simulated images created for video S3. (A) the 2048 x 2048 reference image with 0.7 AR ellipses. (B) One of the 10 simulated 0.7 AR images (512 x512), which is the reference image

from A with the simulated LCTEM noise (8 nm Gaussian blur) added. (C) the 2048 x 2048 reference image with 0.8 AR ellipses. (D) One of the 10 simulated 0.8 AR images (512 x512), which is the reference image from C with the simulated LCTEM noise (8 nm Gaussian blur) added. (E) One frame from the raw **S10** LCTEM video for comparison.

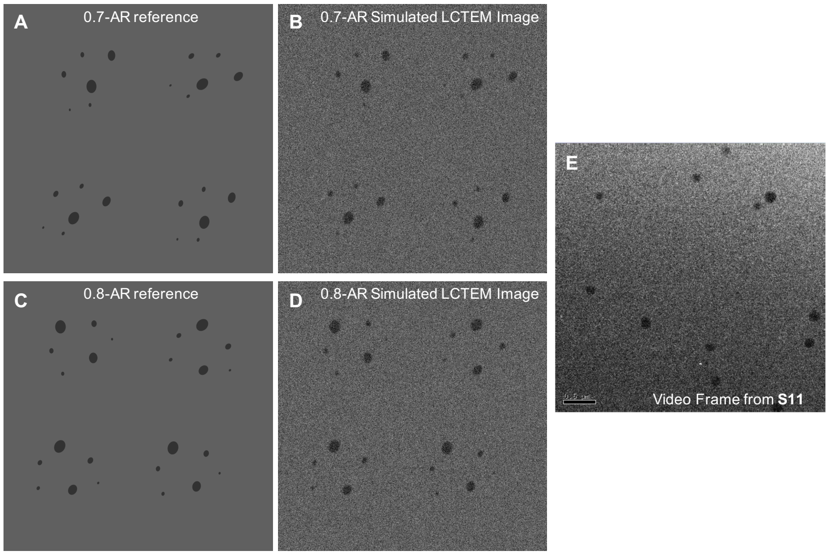

Figure S6. Sample of the simulated images created for video S3. (A) the 2048 x 2048 reference image with 0.7 AR ellipses. (B) One of the 10 simulated 0.7 AR images (512 x512), which is the reference image from A with the simulated LCTEM noise (8 nm Gaussian blur) added. (C) the 2048 x 2048 reference image with 0.8 AR ellipses. (D) One of the 10 simulated 0.8 AR images (512 x512), which is the reference image from C with the simulated LCTEM noise (8 nm Gaussian blur) added. (E) One frame from the raw **S11** LCTEM video for comparison.

METHODS: **MOTA Position and Rotation Tracking Error Measurement (FINAL ERROR Values)**:

1. MOTA tracking algorithm was applied to all 10 of the simulated [and reference] LCTEM images for video **S3** (**Table S5**)
   - The 4 largest 0.7-AR ellipses (70 x100 pixels) and the 4 largest 0.8-AR ellipses (80 x 100 pixels) were tracked in the reference and simulated images
   - For the LCTEM simulated images, the center of mass position error is σ = 1.52 nm
   - For the LCTEM simulated images, the major axis orientation angle error is σ = 10.82°
2. MOTA tracking algorithm was applied to all 10 of the simulated [and reference] LCTEM images for video **S6** (**Table S6**)

- The 4 largest 0.7-AR ellipses (70 x100 pixels) and the 4 largest 0.8-AR ellipses (80 x 100 pixels) were tracked in the reference and simulated images
- For the LCTEM simulated images, the center of mass position error is σ = 2.54 nm
- For the LCTEM simulated images, the major axis orientation angle error is σ = 9.06˚

3. MOTA tracking algorithm was applied to all 10 of the simulated [and reference] LCTEM images for video **S10** (**Table S7**)
    - The 4 largest 0.7-AR ellipses (70 x100 pixels) and the 4 largest 0.8-AR ellipses (80 x 100 pixels) were tracked in the reference and simulated images
    - For the LCTEM simulated images, the center of mass position error is σ = 3.32 nm
    - For the LCTEM simulated images, the major axis orientation angle error is σ = 7.86˚
4. MOTA tracking algorithm was applied to all 10 of the simulated [and reference] LCTEM images for video **S11** (**Table S8**)
    - The 4 largest 0.7-AR ellipses (70 x100 pixels) and the 4 largest 0.8-AR ellipses (80 x 100 pixels) were tracked in the reference and simulated images
    - For the LCTEM simulated images, the center of mass position error is σ = 2.54 nm
    - For the LCTEM simulated images, the major axis orientation angle error is σ = 9.06˚

Table S5. Final center of mass (x,y) position error (σ) and major axis orientation angle error (σ) for the MOTA-extracted trajectories from LCTEM video **S3**.

**Video S3**	**S3_NP_A**	**S3_NP_E**
σ **[x-position] (nm)** from micelle velocity	1.843	2.132
σ **[y-position] (nm)** from micelle velocity	2.191	3.094
σ **[x-position] (nm)** from MOTA tracking error and simulated noise	1.52	1.52
σ **[y-position] (nm)** from MOTA tracking error and simulated noise	1.52	1.52
**σ [x-position] (nm) Total**	**3.363**	**3.652**

**σ [y-position] (nm) Total**	**3.711**	**4.614**
**σ [rotation] (deg.) Total**	**10.82°**	**10.82°**

Table S6. Final center of mass (x, y) position error (σ) and major axis orientation angle error (σ) for the MOTA-extracted trajectories from LCTEM video **S6**.

**Video S6**	**S6_NP_A**	**S6_NP_E**	**S6_NP_F**
**σ [x-position] (nm)** from micelle velocity	1.923	1.402	3.03
**σ [y-position] (nm)** from micelle velocity	3.058	1.949	2.089
**σ [x-position] (nm)** from MOTA tracking error and simulated noise	2.54	2.54	2.54
**σ [y-position] (nm)** from MOTA tracking error and simulated noise	2.54	2.54	2.54
**σ [x-position] (nm) Total**	**4.463**	**3.942**	**5.57**
**σ [y-position] (nm) Total**	**5.598**	**4.489**	**4.629**
**σ [rotation] (deg.) Total**	**9.06°**	**9.06°**	**9.06°**

Table S7. Final center of mass (x, y) position error (σ) and major axis orientation angle error (σ) for the MOTA-extracted trajectories from LCTEM video **S10**.

**Video S10**	**S10_NP_B**	**S10_NP_C**	**S10_NP_D**

σ **[x-position] (nm)** from micelle velocity	1.167	2.238	1.762
σ **[y-position] (nm)** from micelle velocity	2.787	1.57	2.024
σ **[x-position] (nm)** from MOTA tracking error and simulated noise	3.32	3.32	3.32
σ **[y-position] (nm)** from MOTA tracking error and simulated noise	3.32	3.32	3.32
**σ [x-position] (nm) Total**	**4.487**	**5.558**	**5.082**
**σ [y-position] (nm) Total**	**6.108**	**4.89**	**5.344**
**σ [rotation] (deg.) Total**	**7.86°**	**7.86°**	**7.86°**

Table S8. Final center of mass (x, y) position error (σ) and major axis orientation angle error (σ) for the MOTA-extracted trajectories from LCTEM video **S11**.

**Video S11**	**S11_NP_C**
σ **[x-position] (nm)** from micelle velocity	2.091
σ **[y-position] (nm)** from micelle velocity	1.505
σ **[x-position] (nm)** from MOTA tracking error and simulated noise	2.54
σ **[y-position] (nm)** from MOTA tracking error and simulated noise	2.54
**σ [x-position] (nm) Total**	**4.631**
**σ [y-position] (nm) Total**	**4.045**
**σ [rotation] (deg.) Total**	**9.06°**

Section III: Effects of static and dynamic errors on the estimate of the scaling exponent of a diffusing particle.

Static localization error, which stands for the random error in the measurement of an immobilized particle's position, has an impact on the mean squared displacement (MSD) of a diffusing particle [6]

$$MSD(t) = 2Dt + 2\sigma^2 \tag{S.1}$$

where D is the diffusion coefficient in units of L^2/T, $t = n\Delta t$ stands for the time, Δt is the elapsed time between two measurements, and σ is the static localization error, practically expresses the standard deviation in measured positions of an immobile particle. [7,8]
The additional effect of motion blur (dynamical error), wherein a particle's average position over the camera frame interval Δt is measured, has been taken into account and the MSD reads [9,10]

$$MSD(t) = 2Dt + 2\sigma^2 - 4DR\Delta t \text{ , } 0 \leq R \leq 1/4 \tag{S.2}$$

where R is the "motion blur coefficient".

The generalized version of equation (S.2), reads

$$MSD(t) = 2Dt^{\gamma} + 2\sigma^2 - 4DR\Delta t \text{ , } 0 \leq R \leq 1/4 \tag{S.3}$$

Equation (S.3) takes into account a scaling exponent different than one leading either to sub or to super diffusion. The variation of the scaling exponent over time is given by the partial derivative $\partial \log\big(MSD(t)\big) / \partial \log(t)$ which leads to the general relation

$$\gamma(t) = \gamma \frac{1}{1+\frac{\sigma^2}{Dt}-\frac{2R\Delta t}{t}} \tag{S.4}$$

We use equation (S.3) for the estimation of the scaling exponent, the static localization error, and the motion blur. The obtained value of the static error taken by equation (S.3) is called σ^2_{fit} and this value is compared to the square of camera's errorm, σ^2_{cam}. The closer to one the value $\sigma^2_{cam}/\sigma^2_{fit}$ the better the description of the static error on micelle's motion. As this value becomes smaller the less the influence of the static error on particle's erratic motion. We fit equation (S.3) for all the measured trajectories of micelles under different dose rates in x-and y-axes. We should notice that equation (S.1) has been used

twice, namely, x-trajectory of the S6_2_A micelle, and for the y-trajectory of the S11_NP_C micelle. Equation (S.1) for the rest of the recorded trajectories gives negative values of the square of the static error, which has not has a physical meaning.

Table S9: Low dose rate 1.6 $e^{-}/Å^{2}s$, unconstrained fitting by using equation (S.3) delivers the scaling exponent, γ, the square of the fitting static error, $σ^2_{fit}$, the blur coefficient, R, the diffusion coefficient, D. When equation (S.2) is used for fitting then we notice γ=---, which means that the actual value of the scaling exponent is one, γ=1. Furthermore, if the blur coefficient is not used for fitting then we notice R=----. We keep the same description in Tables S10 and S11.

	Fitting with Equation (S.3)	
	x-axis	y-axis
S10_NP_B	γ=0.642±0.016<br>D=730.6±0.5<br>R=0.25±0.016<br>$σ^2_{fit}$=52±10^{-5}<br>$σ^2_{cam}$=20<br>$σ^2_{cam}$/ $σ^2_{fit}$=**0.39**	γ=---------<br>D=544.6±0.5<br>R=0.25±0.011<br>$σ^2_{fit}$=89±10^{-5}<br>$σ^2_{cam}$=37<br>$σ^2_{cam}$/ $σ^2_{fit}$=**0.42**
S10_NP_C	γ=----------<br>D=444±0.527<br>R=0.25±0.147<br>$σ^2_{fit}$=172±2x10^{-4}<br>$σ^2_{cam}$=31<br>$σ^2_{cam}$/ $σ^2_{fit}$=**0.18**	γ=1.000±0.012<br>D=279±0.522<br>R=0.25±0.032<br>$σ^2_{fit}$=208±6x10^{-5}<br>$σ^2_{cam}$=24<br>$σ^2_{cam}$/ $σ^2_{fit}$=**0.11**
S10_NP_D	γ=0.767±0.009<br>D=541±0.504<br>R=0.25±0.016<br>$σ^2_{fit}$=41±1x10^{-5}<br>$σ^2_{cam}$=26<br>$σ^2_{cam}$/ $σ^2_{fit}$=**0.63**	γ=0.84±0.008<br>D=447±0.504<br>R=0.25±0.014<br>$σ^2_{fit}$=63±1x10^{-5}<br>$σ^2_{cam}$=28.55<br>$σ^2_{cam}$/ $σ^2_{fit}$=**0.45**

For the lowest dose rate, the mean value of $σ^2_{cam}$/ $σ^2_{fit}$ in x-axis is 0.4, and in y-axis is 0.33. It means that the static error as well the motion blur contribute to the particle's motion roughly about 40% and 33% in each axis respectively . However, these values are accompanied by scaling exponents less than one in three cases while in the rest three the scaling exponent is one. By using equation (S.4) the variation of the

scaling exponent is illustrated in Figure S7. All motions except one start superdiffusive and then converge to a constant value describing sub or normal diffusion.

There is no a single trajectory where its anomalous character can be attribute to the static error and/or motion blur.

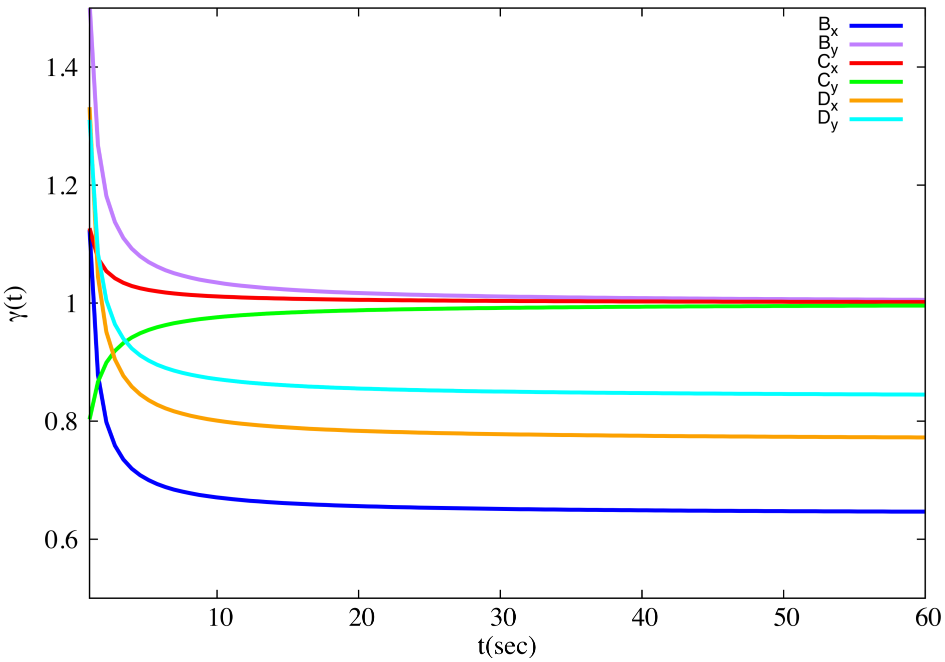

Figure S7. Variation of the scaling exponent over time for the low dose rate according to equation (S.4).

Initially, micelles undergo a super diffusive motion which slows down over time and converges either to sub-diffusion or to normal diffusion in the long time limit. Notice that the normal diffusion here is described by scaling exponents a bit higher than one, close to 1.1.

Table S10: The same as Table S9 for dose rate 2.6 $e^-/Å^2$.

	Fitting with equation (S.3)	
	x-axis	y-axis
S6_NP_A	γ=---------<br>D=356±0.52<br>R=-----------<br>σ^2_{fit}=226±72<br>σ^2_{cam}=20<br>$\sigma^2_{cam}/\sigma^2_{fit}$=**0.09**	γ=---------<br>D=726±0.5<br>R=0.06±0.0001<br>σ^2_{fit}=85±10^{-5}<br>σ^2_{cam}=31<br>$\sigma^2_{cam}/\sigma^2_{fit}$=**0.37**
S6_NP_E	γ=0.744±0.012	γ=------------

	D=650±0.52<br>R=0.03±0.0.03<br>σ^2_{fit}=361±2x10^{-5}<br>σ^2_{cam}=15.5<br>σ^2_{cam}/ σ^2_{fit}=**0.04**	D=300±0.513<br>R=0.249±0.090<br>σ^2_{fit}=528±2x10^{-5}<br>σ^2_{cam}=20<br>σ^2_{cam}/ σ^2_{fit}=**0.04**
S6_NP_F	γ=1.320±0.048<br>D=430±0.520<br>R=----------<br>σ^2_{fit}=298±83<br>σ^2_{cam}=31<br>σ^2_{cam}/ σ^2_{fit}=**0.10**	γ=1.254±0.026<br>D=765±0.513<br>R=0.0±0.049<br>σ^2_{fit}=199±3x10^{-5}<br>σ^2_{cam}=21.42<br>σ^2_{cam}/ σ^2_{fit}=**0.11**
S11_NP_C	γ=1.280±0.033<br>D=314±0.546<br>R=----------<br>σ^2_{fit}=179±35<br>σ^2_{cam}=21.45<br>σ^2_{cam}/ σ^2_{fit}=**0.12**	γ=-----------<br>D=264.5±0.520<br>R=----------<br>σ^2_{fit}=399±46<br>σ^2_{cam}=16.36<br>σ^2_{cam}/ σ^2_{fit}=**0.04**

Increasing the dose rate the mean value of σ^2_{cam}/ σ^2_{fit} in x-axis is 0.09, and in y-axis is 0.14. The contribution of the static localization error and motion blur on micelles motion is very small and their anomalous character cannot be attributed to these effects. The same is true as we increase more the dose rate, Table S11, where the mean value of σ^2_{cam}/ σ^2_{fit} in x-axis is 0.07, and in y-axis is 0.25.

Table S11: The same as Table S9 for dose rate 5.6 e^-/Å^{2}s.

	Fitting equation (S.3)	
	x-axis	y-axis
S3_NP_A or<br>V2_2_A	γ=1.173±0.050<br>D=494±0.511<br>R=-----------<br>σ^2_{fit}=146±53<br>σ^2_{cam}=13.18<br>σ^2_{cam}/ σ^2_{fit}=**0.09**	γ=---------<br>D=598±0.517<br>R=0.214±0.044<br>σ^2_{fit}=254±3x10^{-5}<br>σ^2_{cam}=13.77<br>σ^2_{cam}/ σ^2_{fit}=**0.05**
S3_NP_E or<br>V2_2_E	γ=1.257±0.049<br>D=287±0.525	γ=1.118±0.060<br>D=683±0.530

	R=----------------<br>σ^2_{fit}=266±54<br>σ^2_{cam}=13.34<br>$\sigma^2_{cam}/\ \sigma^2_{fit}$=**0.05**	R=0.248±0.070<br>σ^2_{fit}=590±6x10^{-5}<br>σ^2_{cam}=13.77<br>$\sigma^2_{cam}/\ \sigma^2_{fit}$=**0.02**

By analyzing the effects of static localization error and of motion blur on micelles anomalous motion for these sets of experiments one can safely conclude that their contribution on particles motion is negligible. Furthermore, there is no single experiment where its anomalous character can be solely associated either to static error and/or of motion blur. We see that the lower the dose rate the higher the contribution of these factor, probably connected to contrast effects, without of course to be considered as dominant sources of anomalous diffusion.

Section IV: Preliminary analysis of raw data

The raw data monitor the coordinates (*x*- and *y*- axes expressed with respect to the lab frame) of each micelle in the course of time with nanometer resolution.

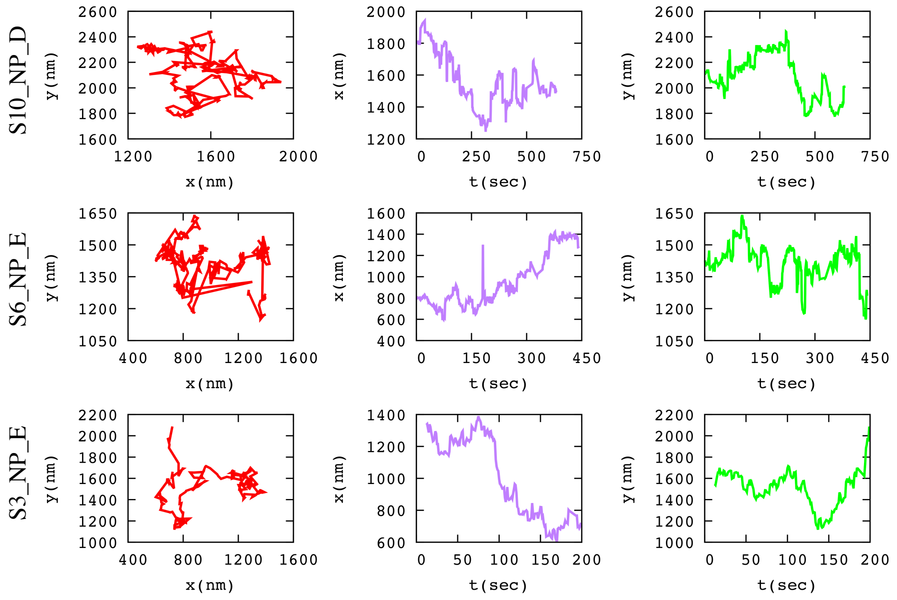

Figure S8. Trajectories traced by micelles within LCTEM environment. Colour code: red for lateral motion of a micelle, purple for the variation in the course of time of the x-coordinate of the micelle, and green for the y-coordinate. All coordinates are expressed in the lab-frame. The depicted trajectories correspond to three different radiation dose rates; id. S10_NP_D for 1.6 e-/Å^{2}s , id. S6_NP_E for 2.6 e-/Å^{2}s, and id. S3_NP_E for 5.6 e-/Å^{2}s.

For each micelle we construct the following time series: $\{x_n\}, \{y_n\}$ with n=1,2,…,N, where N is the total number of data points, which correspond to the recorded coordinates {*x*(t), *y*(t)}. If τ is the minimum time lag between two consecutive measurements and T the total length of the recorded trajectory then T=N τ. Figure S8 shows the lateral displacement of a micelle (red colour), as well the evolution in time of each one of the recorded coordinates (*x* and *y*). One can easily notice that the motion depicting either the lateral displacement or the jumps taken in *x*- or *y*-axes contains some big jumps interrupted by periods of constrained motion in a small area or by periods of complete immobilization. A very first analysis of such a behaviour can be made by examining the probability of length steps taken by the micelle, P(l). The length step is given by the absolute value of the difference between two consecutive points either in *x* or in *y* axis, while, for lateral displacements the length is $l_i = \sqrt{(x(i+1) - x(i))^2 + (y(i+1) - y(i))^2}$. We define the random variable ξ(i)=l(i)/<l>, where <l> is the mean, and we estimate the probability distribution P(ξ). Figure S9 shows the probability distribution P(ξ) versus ξ for some micelles under different radiation dose conditions.

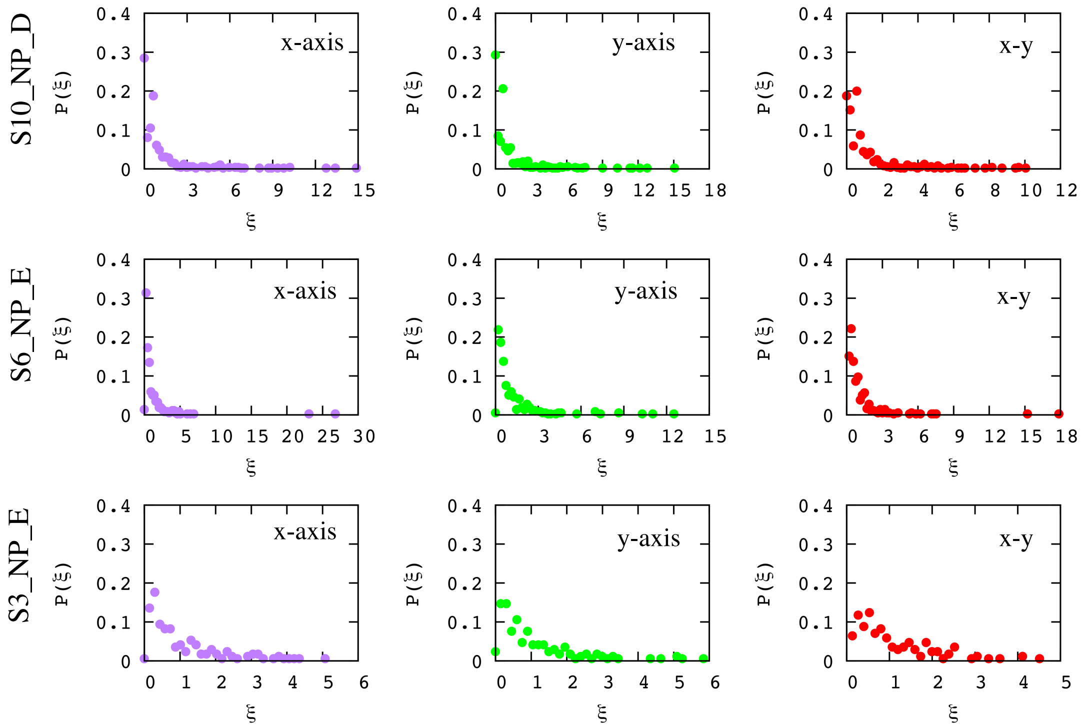

Figure S9. Probability distribution P(ξ) versus ξ for movements either in *x* or in *y*-axis or lateral displacements. Colour code and i.d. of micelles are exactly the same as those illustrated in Figure S8.

For 1.6 e-/Å^{2}s radiation dose rate, the probability P(ξ) poses its maximum at value ξ=0, which underlines immobilization of the motion and it is a characteristic feature of Continuous Time Random Walk (CTRW)[9]. Interestingly, the same probability for lateral displacements attains its maximum again at ξ=0 but the

value of the probability is significant reduced with respect to the corresponding probabilities for motion in *x*- and *y*- axes. This finding indicates that, there exist time periods where the motion is immobilized on the surface but there also exist time periods where the motion is immobilized in one axis and is free to move in the second one. For 2.6 e-/Å^{2}s radiation dose rate, (i.d. S6_NP_E), the maximum of the probability distribution moved from zero to a value very close to it. A first sign of the role of the radiation dose is observed, the higher the dose rate the lower the number of immobilization events.[8] For lateral displacements the probability for immobilization events is zero. The same behaviour is also observed for 5.6 e-/Å^{2}s radiation dose rate. In general, as the radiation dose increases, the probability of finding immobilization events decreases.

The shape of the probability of length steps suggests that CTRW[9] is likely the stochastic mechanism. CTRW is an ageing random process, which means that the choice of the initial starting point of the process determines the behaviour of the mean square displacement (MSD). Or in other words: if CTRW is the underlying random process then by increasing the length of the analysing time series and by keeping constant the time lag between two points, more and more trapping events are encounter, changing thus the behaviour of the (MSD). It has been pointed out that MSD as function of time for constant lag, Δ, scales as[11,12]

$$< x_{\Delta}^{2}(t) > \sim \frac{\Delta}{t^{1-\gamma}} \qquad \text{(S.5)}$$

which for discrete time series (individual trajectories and for fixed time lag, Δ) is calculated as a time average (TA-MSD) being function of the length of trajectory, *t*, and it reads

$$MSD_{\Delta}(t) = \overline{x_{\Delta}(t)} = \frac{1}{M-\Delta}\sum_{n=1}^{M-\Delta}\{x(n+\Delta) - x(n)\}^2 \qquad \text{(S.6)}$$

where, $t = M\Delta$ is the running length, and $T = N\Delta$ is the total length of the trajectory, $M \leq N$. Using equation (S.6) we take as minimum length $t = T/2$. The behavior of $MSD_{\Delta}(t)$ for motions in either *x*- or *y*- axes or for lateral motion is illustrated in Figure S10a.

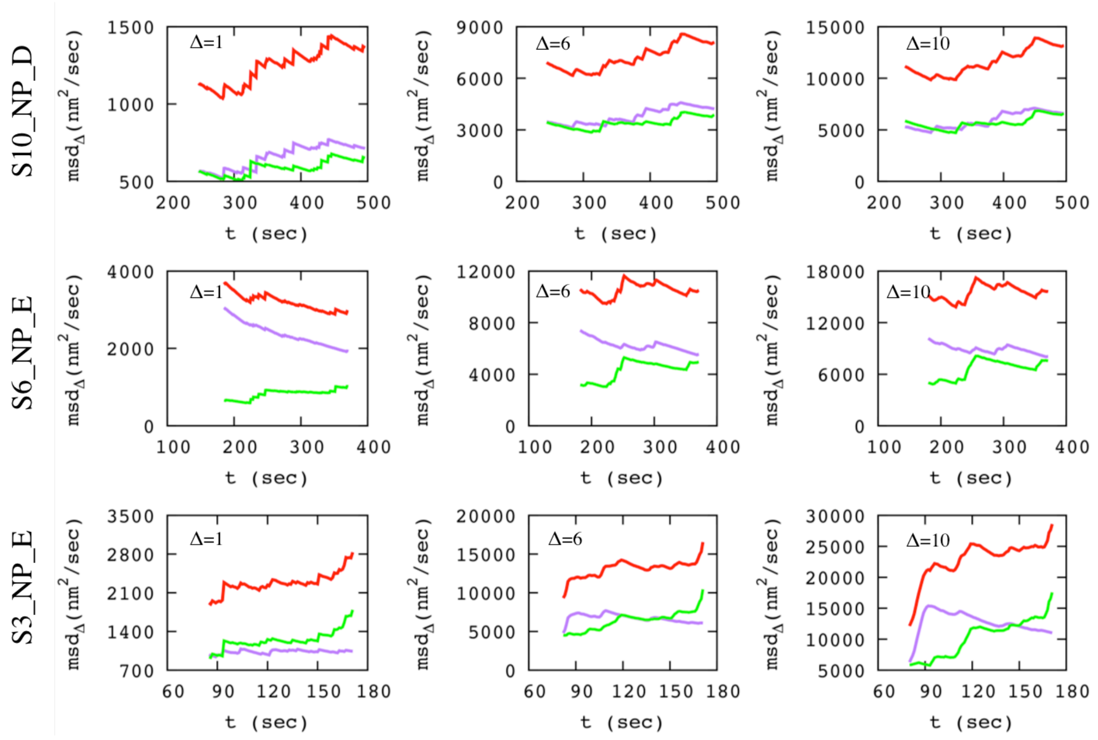

Figure S10a. $MSD_{\Delta}(t)$ for three different constant time lags, Δ=1, Δ=6, and Δ=10 sec, for random movements in either *x*- or *y*-axes or for lateral displacements. Three different micelles each one under different radiation dose rate have been considered. The colour code is the same as for Figures S8 and S9.

The behavior of $MSD_{\Delta}(t)$ does not give a clear evidence of ageing effects, Figure S10a. There are cases where MSD decreases as *t* increases, in some others MSD remains pretty much constant as function of time, and there are also cases where MSD increases as function of the running time *t*. Increasing or decreasing trends depend on the actual distribution of the traps in the trajectory. For traps distributed at the beginning of the running trajectory, the overall trend increases, while for those distributed at the end of the trajectory the trend decreases. Furthermore, for single trajectories, the TA-MSD presents rough behavior; a smoothing is necessary and can be made through the ensemble average of all available trajectories. Here, each micelle is different from the others and the trajectories are not of the same length, so ensemble average cannot be taken.

The distribution of trapping events of a given sequence of raw data can be further connected to the effects of the radiation dose rate since the latter continuously provides energy to the system. We further explore this point by checking ageing effects of the time-reversed time-series. We analyse the same trajectories

presented in Figure S3a, where now the coordinates read, x(j)=x(N-i+1), y(j)=y(N-i+1), for i=1,2,3,....,N, and the results are illustrated in Figure S3b.

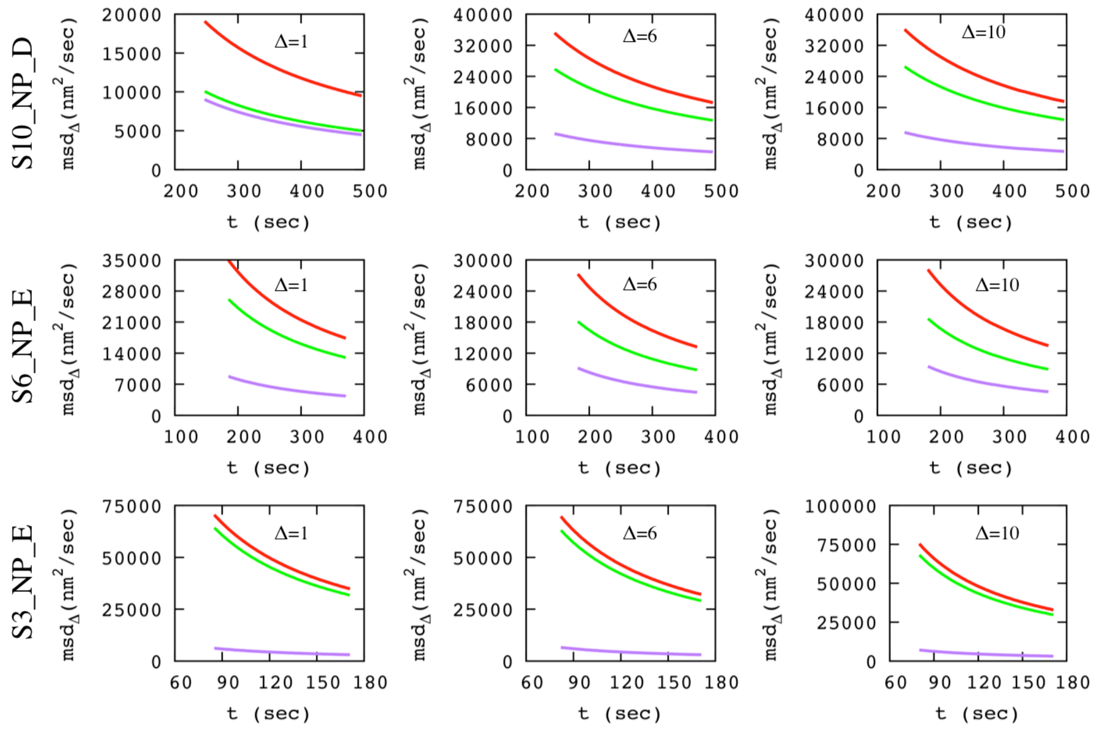

Figure S10b. Time-reversed raw data analysis. $MSD_\Delta(t)$ for three different constant time lags, Δ=1, Δ=6, and Δ=10 sec, for random movements in either *x*- or *y*-axis or for lateral displacements. Three different micelles under different radiation dose rates have been considered. The colour code as for Figures S8 and S9.

For all micelles and for all radiation dose rates and for fixed lag time, $MSD_\Delta(t)$ is a decreasing function of the running time, *t*, which is an evidence of ageing. The time-reversed time series of a LCTEM experiment underline the e-beam effect on the ability of the environment to operate as trap for a micelle. The number of trapping events is decreasing and this is in agreement with the findings depicted in Figure S9.

CTRW is a random process characterized by long-tailed distributions of waiting times, which has the form $\psi(t) \sim t^{-1-\beta}$, with 0< β <1, if we consider waiting times with infinity mean, or $1 < \beta < 2$ considering waiting times with infinity variance.[13] This probability distribution corresponds to time periods between successive jumps and can be easily obtained by using the raw data. The strict condition for the estimation of $\psi(t)$ considers as steps taken by the particle only those with length different than zero. This condition can relax considering also as steps contributing to waiting times those with displacements much smaller than the

size of the particle. We have used as cut-off a value of $0.1std\{l(i)\}$ for each micelle and the estimated waiting times probabilities are depicted in Figure 3 of the main text.

For low rate dose (1.6 e-/Å^{2}s), all analysed raw data (lateral displacement, in red, or independent movements in either *x*- or *y*-axis, purple and green respectively) for the micelle with id. S10_NP_D give a scaling exponent in the range $0.64 \leq \beta \leq 0.78$, and $0.55 \leq \beta \leq 1.0$ for dose rate (2.6 e-/Å^{2}s), for the highest dose rate $\psi(t)$ is not indicative.

Probability distribution of waiting times for low and intermediate rate doses distinguishes anisotropy in motion in *x*- and *y*-axes. If CTRW were the driving stochastic process then the second moment or Mean Square Displacement would scale with the same exponents as $\psi(t)$ does. However, by comparing the above exponents with the values of γ_{MSD} for the same micelles listed in Table 1 of the main text we see that these values are different. CTRW is NOT the stochastic process that governs the motion because scaling of distributions of waiting times are not coincide with scaling of MSD and the recorded time series do not present ageing.

Section V: Analysing Rotational movements

The available rotational time series have been analysed by using equation (5) of the main text. Moments up to third order including fractional ones have been obtained. All moments have been fitted by equation (3) of the main text. For all moments, the obtained exponents are zero or very close to it indicating thus stationary time series. The physical interpretation of such a time series likely points to a micelle that rotates randomly around a fixed point. Some representative examples are illustrated in the Figures S11a to S11c

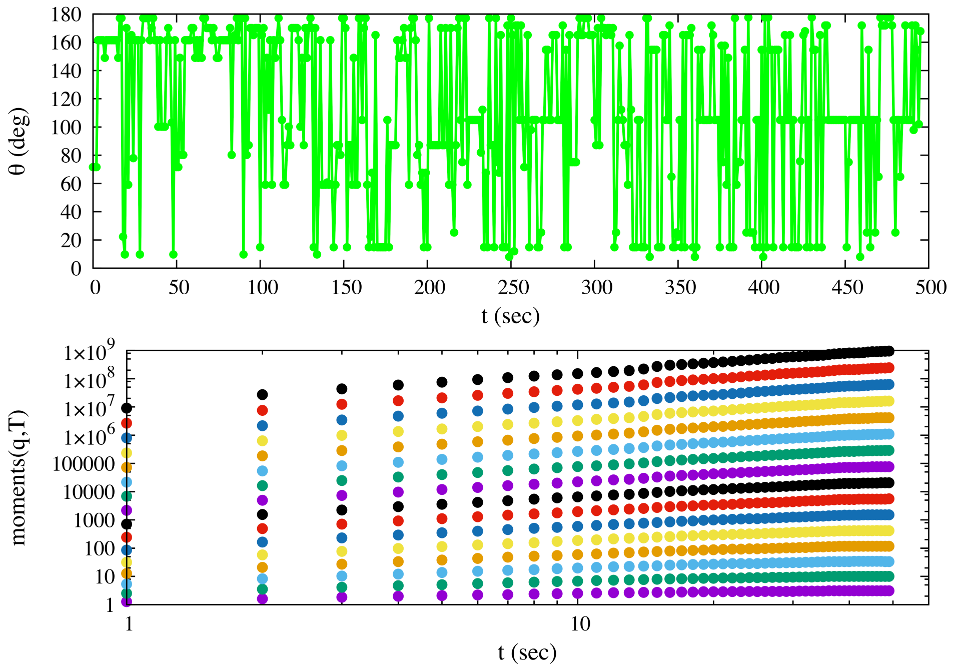

Figure S11a. Orientation data for one individual micelle (micelle id: S10_NP_D). (A) variation of the angle formed between the main axis of the ellipse with the *x*-axis of the laboratory frame of reference versus the elapsed time. (B) Moments up to fourth order obtained by ADOMA, equation (5), versus the lag time in log-log scale. From bottom to top moments increase by 0.25: the fourth from bottom (orange) is the mean, the eight from bottom (black) is the MSD. All moments present a very small slope (scaling exponent), smaller than 0.1 highlighting thus a weak-stationary process.

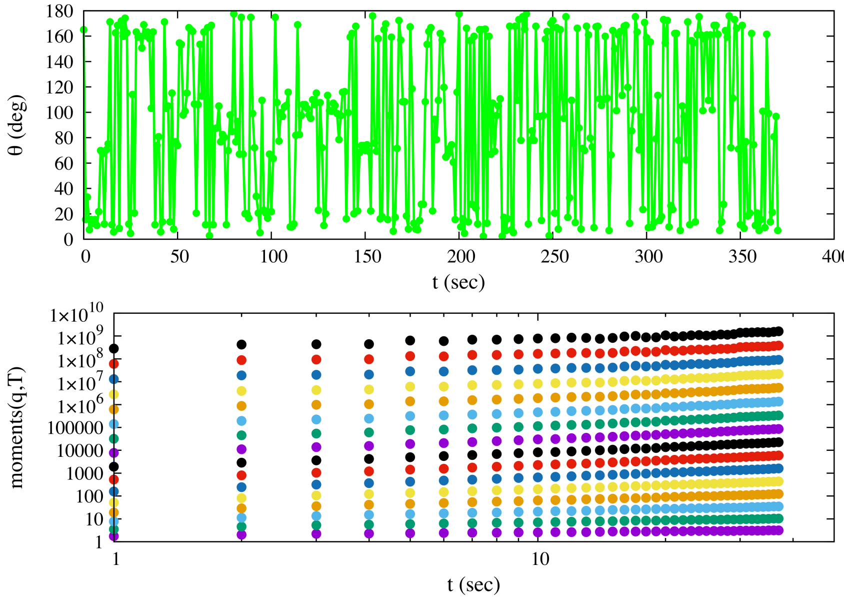

Figure S11b. Orientation data for one individual micelle (micelle id: S6_NP_E). (A) variation of the angle formed between the main axis of the ellipse with the *x*-axis of the laboratory frame of reference versus the elapsed time. (B) Moments up to fourth order obtained by ADOMA versus the lag time in log-log scale. From bottom to top moments increase by 0.25: the fourth from bottom (orange) is the mean, the eight from bottom (black) is the MSD. All moments present a very small slope (scaling exponent), smaller than 0.1 highlighting thus a weak-stationary process.

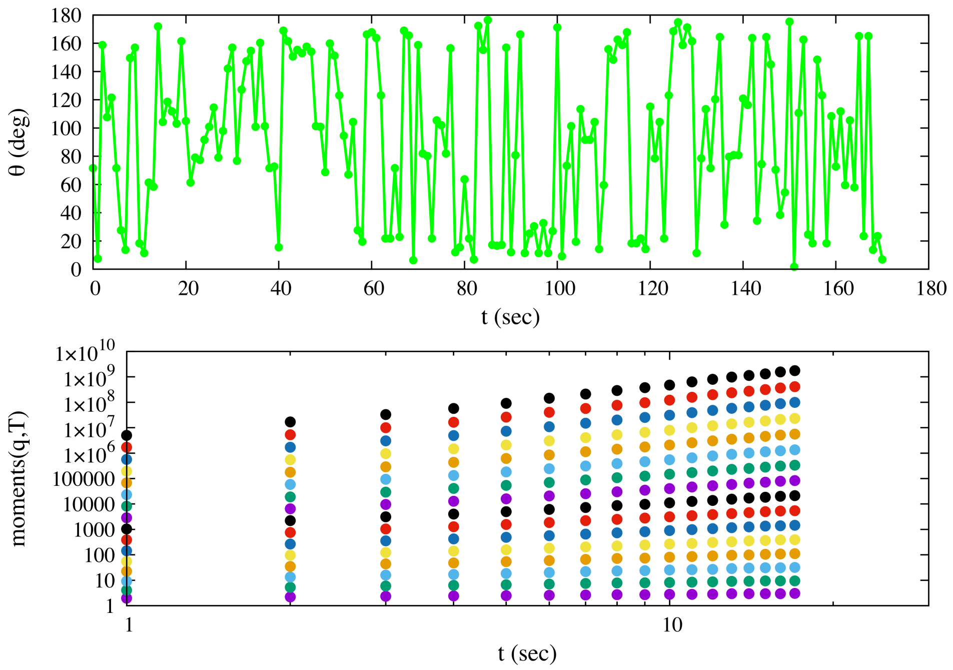

Figure S11c. Orientation data for one individual micelle (micelle id: S3_NP_E). (A) variation of the angle formed between the main axis of the ellipse with the x-axis of the laboratory frame of reference versus the elapsed time. (B) Moments up to fourth order obtained by ADOMA versus the lag time in log-log scale. From bottom to top moments increase by 0.25: the fourth from bottom (orange) is the mean, the eight from bottom (black) is the MSD. All moments present a very small slope (scaling exponent), smaller than 0.1 highlighting thus a weak-stationary process.

Section VI: Analysing variations in time of micelles size

The examined micelles are of elliptical shape (aspect ratio ~0.7). It is also available the variation of the aspect ratio over time for each one of the analysed micelles. We obtain the moments for each one of these time series by applying equation (5) of the main text, and in the following we demonstrate the first, and the second moment, as well the variance for each micelle and for all dose rates. All depicted measures have not have time dependency, indicating thus that aspect ratio does not change in time.

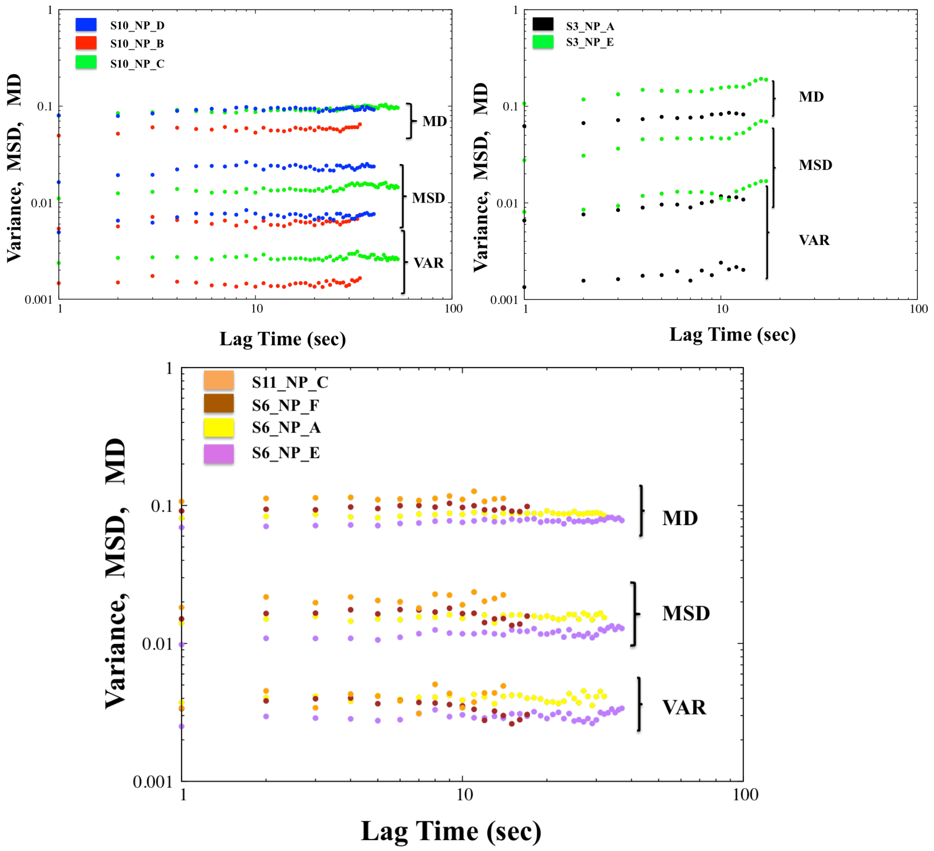

Figure S12. The variance and the first two moments (first and second) of the aspect ratio as a function of the lag time for different micelles under different irradiation conditions. In all cases, variance and moments are constant in time indicating that micelles retain their initial elliptical shape during the experiment.

References:

1 Park, C.; Woehl, T. J.; Evans, J. E.; Browning, N. D. Minimum Cost Multi-Way Data Association for Optimizing Multitarget Tracking of Interacting Objects. *IEEE Trans. Pattern Anal. Mach. Intell.*, **2015**, *37*, 611–624.

2. Vo, G.; Park, C. Robust regression for image binarization under heavy noise non-uniform background. *Pattern Recogn.*, **2018**, *81*, 224-239.

3. Parent, L. R.; Bakalis, E.; Ramirez-Hernandez, A.; Kammeyer, J. K.; Park, C.; de Pablo, J.; Zerbetto, F.; Patterson, J. P.; Gianneschi, N. C. Directly Observing Micelle Fusion and Growth in Solution by Liquid Cell Transmission Electron Microscopy. *J. Am. Chem. Soc.*, **2017**, *139*, 17140–17151.

4. Parent, L. R.; Bakalis, E.; Proetto, M.; Li, Y.; Park, C.; Zerbetto, F.; Gianneschi, N. C. Tackling the Challenges of Dynamic Experiments Using Liquid-Cell Transmission Electron Microscopy. *Acc. Chem. Res.*, **2018**, *51*, 3–11.

5 Boothroyd, C. B.; Kasama, T.; Dunin-Borkowski, R. E. Comparison of approaches and artefacts in the measurement of detector modulation transfer functions. *Ultramicroscopy*, **2013**, *129*, 18–29.

6. Martin, D.; Forstner, M.; Kas, J. Apparent Subdiffusion Inherent to Single Particle Tracking. *Biophys. J.*, **2002**, *83*, 2109.

7. Thompson, R.; Larson, D.; Webb, W. Precise Nanometer Localization Analysis for Individual Fluorescent Probes. *Biophys. J.*, **2002**, *82*, 2775.

8. Ober, R.; Ram, S.; Ward, W. Localization Accuracy in Single_Molecule Microscopy. *Biophys. J.*, **2004**, *86*, 1185.

9. Savin, T.; Doyle, P. S. Static and Dynamic Errors in Particle Tracking Microrheology. *Biophys. J.*, **2005**, *88*, 623.

10. Berglund, A. J. Statistics of camera-based single-particle tracking. *Phys. Rev. E*, **2010**, *82*, 011917.

11. Metzler, R.; Jeon, J-H.; Cherstvy, A. G.; Barkai, E. Anomalous diffusion models and their properties: non-stationarity, non-ergodicity, and ageing at the centenary of single particle tracking. *Phys.Chem.Chem.Phys.*, **2014**, *16*, 24128.

12. Lubelski, A.; Sokolov, I. M.; Klafter, J. Nonergodicity Mimics Inhomogeneity in Single Particle Tracking. *Phys. Rev. Lett.*, **2008**, *100*, 250602.

13. Weiss, G. H.; Rubin, R. J. Random Walks: Theory And Selected Applications, *Adv. Chem. Phys.*, **1983**, *Vol. LII*, 363-505.
